# Acetate Degradation at Low pH by the Moderately Acidophilic Sulfate Reducer *Acididesulfobacillus acetoxydans* gen. nov. sp. nov.

**DOI:** 10.3389/fmicb.2022.816605

**Published:** 2022-03-04

**Authors:** Irene Sánchez-Andrea, Charlotte M. van der Graaf, Bastian Hornung, Nicole J. Bale, Monika Jarzembowska, Diana Z. Sousa, W. Irene C. Rijpstra, Jaap S. Sinninghe Damsté, Alfons J. M. Stams

**Affiliations:** ^1^Laboratory of Microbiology, Wageningen University & Research, Wageningen, Netherlands; ^2^Laboratory of Systems and Synthetic Biology, Wageningen University & Research, Wageningen, Netherlands; ^3^Department of Marine Microbiology and Biogeochemistry, NIOZ Royal Netherlands Institute for Sea Research, Den Burg, Netherlands; ^4^Department of Earth Sciences, Faculty of Geosciences, Utrecht University, Utrecht, Netherlands; ^5^Centre of Biological Engineering, University of Minho, Braga, Portugal

**Keywords:** acid rock/mine drainage, acidophiles, sulfate-reducing bacteria, *Acididesulfobacillus*, *Desulfosporosinus*, *Desulfitobacterium*, acetate oxidation, bioremediation

## Abstract

In acid drainage environments, biosulfidogenesis by sulfate-reducing bacteria (SRB) attenuates the extreme conditions by enabling the precipitation of metals as their sulfides, and the neutralization of acidity through proton consumption. So far, only a handful of moderately acidophilic SRB species have been described, most of which are merely acidotolerant. Here, a novel species within a novel genus of moderately acidophilic SRB is described, *Acididesulfobacillus acetoxydans* gen. nov. sp. nov. strain INE, able to grow at pH 3.8. Bioreactor studies with strain INE at optimum (5.0) and low (3.9) pH for growth showed that strain INE alkalinized its environment, and that this was more pronounced at lower pH. These studies also showed the capacity of strain INE to completely oxidize organic acids to CO_2_, which is uncommon among acidophilic SRB. Since organic acids are mainly in their protonated form at low pH, which increases their toxicity, their complete oxidation may be an acid stress resistance mechanism. Comparative proteogenomic and membrane lipid analysis further indicated that the presence of saturated ether-bound lipids in the membrane, and their relative increase at lower pH, was a protection mechanism against acid stress. Interestingly, other canonical acid stress resistance mechanisms, such as a Donnan potential and increased active charge transport, did not appear to be active.

## Introduction

Acid rock and acid mine drainage (ARD and AMD) environments are among the most extreme habitats on Earth due to their high acidity and high metal concentrations. Acid drainage is generated by the oxidative dissolution of metal sulfides to sulfuric acid and dissolved metals upon exposure to water and oxidants such as oxygen (O_2_) or ferric iron (Fe^3+^; Sand et al., 2001; Johnson and Hallberg, 2005). This can be triggered naturally (ARD) or by mining operations (AMD; Lottermoser, 2010). The Tinto River, which flows through the massive sulfide deposits of the Iberian Pyrite Belt (IPB; Huelva, Southwestern Spain), is one of the best-known examples of ARD. Although mining activity reportedly occurred in the IPB already >5,000 years ago, the acid drainage does not exclusively originate on the surface by mining activities but also in the subsurface, as a result of chemolithotrophic microbial activity (Amils et al., 2011).

Microorganisms thriving in acid drainage environments are polyextremophiles, as they need to withstand high acidity (the mean pH of the water column of the Tinto River is 2.3), extremely high metal concentrations (e.g., Fe 2.26 g/L, Cu 0.11 g/L, and Zn 0.24 g/L; López-Archilla et al., 2001) and high salinity due to high sulfate concentrations. Although much emphasis has been placed on the extreme acidophiles involved in the iron cycle and the oxidative part of the sulfur cycle, studies of anoxic Tinto River sediments showed the importance of more moderately acidophilic microorganisms, most notably acidophilic sulfate-reducing bacteria (SRB; Sánchez-Andrea et al., 2011, 2012b). SRB have been shown to have an attenuating effect on the extreme characteristics of the Tinto River through the formation of sulfide (biosulfidogenesis), enabling the precipitation of metals as metal sulfides, and the consumption of protons required for sulfate reduction at low pH, resulting in alkalinization (Sánchez-Andrea et al., 2012a). In specific layers of Tinto River sediments, a higher pH (up to 6.2), a lower redox potential, and lower sulfate and metal concentrations were accompanied by a higher abundance of SRB from the family *Peptococcaceae* (Sánchez-Andrea et al., 2012a), representing up to 40% of the total cell counts in the sediment layers. Geomicrobiological studies of abandoned open pit mines filled with AMD revealed that, similar to observations in AMD sediments, biosulfidogenesis by SRB also had an attenuating effect on acidity and metal concentrations in the water column (Wendt-Potthoff et al., 2012; Santofimia et al., 2013; Falagán et al., 2014; Sánchez-España et al., 2020; van der Graaf et al., 2020).

The in-depth study of the physiology and dominant stress resistance mechanisms of acidophilic SRB requires pure culture isolates. So far, only a few moderately acidophilic or acidotolerant SRB species have been described, *Thermodesulfobium narugense* (pH 4.0–6.5; Mori et al., 2003) and *T. acidiphilum* (pH 3.7–6.5; Frolov et al., 2017), both from the genus *Thermodesulfobium* within the family *Thermodesulfobiaceae*; *Desulfothermobacter acidiphilus* (pH 2.9–6.5; Frolov et al., 2018) from the family *Thermoanaerobacteraceae*; and *Desulfosporosinus acididurans* (pH 3.8-7.0; Sánchez-Andrea et al., 2015), *D. acidiphilus* (pH 3.6–5.5; Alazard et al., 2010), and *D. metallidurans* (pH 4.0–7.0; Panova et al., 2021) from the genus *Desulfosporosinus* in the family *Peptococcaceae* of the *Clostridia* class in the *Firmicutes* phylum. Here, we report the characterization of a novel moderately acidophilic SRB from a novel genus, *Acididesulfobacillus acetoxydans* strain INE, enriched from Tinto River sediments (Sánchez-Andrea et al., 2013). We use comparative proteomics and cell membrane lipid analysis performed on cells grown at optimum (5.0) and low (3.9) pH of growth to determine the main strategies enabling *A. acetoxydans* to grow at acidic pH.

## Materials and Methods

### Microorganisms

Strain INE was isolated from an enrichment culture containing acidic sediments from *JL* dam (37.691207 N, 6.560587 W) in the Tinto River basin (southwestern Spain) by incubation with 5 mM of lactate and 10 mM sulfate at pH 4.5 (Sánchez-Andrea et al., 2013). Detailed physicochemical information on the site has been published previously (Sánchez-Andrea et al., 2012a). For comparison purposes, *D. orientis* DSM 765^T^, *Desulfitobacterium dehalogenans* DSM 9161^T^ and *D. acidiphilus* DSM 22704^T^ were obtained from the DSMZ (Braunschweig, Germany). *Desulfosporosinus acididurans* DSM 27692^T^ was taken from the laboratory collection.

### Phenotypic Characterization: Morphology and Physiology

Cell morphology, motility and spore formation were examined by phase contrast microscopy using a Leica DM2000 microscope (Leica, Hesse, Germany). Cell size was determined by SEM microscopy. To this end, samples were fixed by immersion in glutaraldehyde (2.5%) for 2 h, and subsequently washed twice in sodium cacodylate buffer (0.2 M, pH 7.1). Samples were dehydrated in a graded series (10%, 30%, 50%, 70%, 90%, and 100%) of ethanol/water mixtures, leaving them 20 min in each mixture. After dehydration, samples were critical point dried and mounted on stubs. After gold shadowing, samples were examined in a Phillips XL30 EDAX DX4i SEM (Philips, Netherlands). The lengths and widths of several cells were measured, and mean dimensions recorded. Gram staining was performed according to standard procedures (Doetsch, 1981), and Gram-structure was checked by mixing cells with a drop of 3% (w/v) solution of KOH.

Gram-structure was further checked by TEM microscopy. For this, cells were pelleted, incubated in fixative solution (2.5% glutaraldehyde, 2% paraformaldehyde in a 0.1 M phosphate citrate buffer pH 5.0) for 1 h at room temperature, washed twice in 0.1 M phosphate citrate buffer (wash buffer), resuspended in 100 ml 2% gelatin in 0.1 M phosphate citrate buffer and incubated at 4°C until gelatin solidified. The gelatin pellet was incubated for 15 min at room temperature in fixative solution, cut into small pieces (maximum 0.5 cm^3^), and incubated for 30 min at room temperature. Gelatin sections were washed six times with wash buffer, stained and fixed in a 1% osmium tetroxide solution for 1 h at room temperature. The samples were then washed three times in distilled water, dehydrated in successive ethanol incubations (30%, 50%, 70%, 80%, 90%, and 96%), with a final dehydration step in 100% ethanol. Samples were embedded in Spurr epoxy resin as described previously (Spurr, 1969), cut into ultrathin sections with a Leica EM UC7 ultramicrotome (Leica, Wetzlar, Hesse, Germany), and poststained with uranyl-acetate and lead citrate. Samples were imaged using a JEOL JEM-1400 series 120 kV TEM (JEOL, Tokyo, Japan).

Catalase activity was determined by reaction with 15% (w/v) solution of H_2_O_2_. An oxidase test was performed with a filter impregnated in 1% (w/v) solution of tetramethyl-*p*-phenylenediamine in dimethyl sulfoxide (Sigma-Aldrich, St. Louis, MO). Indole and urease formation as well as gelatin and esculin hydrolysis were determined with API® 20A (bioMérieux, France) according to manufacturer’s instructions. Analysis of respiratory quinones of biomass grown on glycerol was carried out by the DSMZ Deutsche Sammlung von Mikroorganismen und Zellkulturen GmbH (Braunschweig, Germany; Tindall, 1990a,b).

Growth experiments were performed in triplicate, using 120 ml-serum bottles as described elsewhere (Sánchez-Andrea et al., 2013). Growth conditions were pH 5.0 and *T_a_* = 30°C, unless indicated otherwise. Growth was monitored by measuring optical density at 600 nm (OD_600_) with a spectrophotometer (U-1500 Hitachi, Tokyo, Japan). Soluble substrates and intermediates (sugars and volatile fatty acids) were measured using a thermo electron spectrasystem high performance liquid chromatography (HPLC) equipped with an Agilent Metacarb 67H column. Gaseous compounds (H_2_) were analyzed using a Shimadzu GC-2014 Gas Chromatograph equipped with a Mol sieve 13X column. Sulfide was measured photometrically with the methylene blue method (Cline, 1969). Different electron donors and acceptors were tested at final concentrations of 5 or 10 mM, respectively, except sulfite, which was tested at 5 mM. Electron donors and acceptors were added to the media from sterile 1 M stock solutions. When electron donors were tested, sulfate (10 mM) was used as acceptor and when acceptors were tested, glycerol (5 mM) was used as donor. Growth was also studied at different incubation temperatures (from 10°C to 45°C), pH (from 3.0 to 7.5), salinity (up to 0.5 M NaCl), oxygen concentrations, and with different reducing agents: L-cysteine, sodium sulfide, titanium citrate, and FeCl_2_ (2 mM). Sulfate reduction rates were determined during the exponential phase of the cultures grown between pH 4 and 7 at 30°C.

### Bioreactor Cultivation

Bioreactor cultivation was performed in duplicate in two pH-controlled batch reactors at pH 3.9 and pH 5.0. Basal medium was supplemented with 0.1 g yeast extract L^−1^, 5 mM glycerol, 10 mM sulfate, and 2 mM L-cysteine as reducing agent. Glass reactors (Applikon, Netherlands) of 5 L volume capacity filled with 4 L of medium were operated with an ADI 1010 Bio-controller and the ADI 1025 Bio-console (Applikon). Autoclavation of the whole system enabled reactor operation under sterile conditions. Stirring speed was 50 rpm, temperature was maintained at 30°C, and pH was maintained at pH 5.0 or pH 3.9 by addition of 0.5 M HCl. The growth rates at pH 3.9 and pH 5.0 were calculated from semi-logarithmic plots of changes in OD_600_ values against time at 30°C. Values were obtained by implementing the modified Gompertz model (Zwietering et al., 1990). Biomass for lipid and proteome analysis was obtained from the reactors at 80% of maximum OD_600_.

### Membrane Lipid Analysis

For comparison of the cellular fatty acid composition all strains were grown at their optimum pH in the medium described elsewhere (Sánchez-Andrea et al., 2013). Bicarbonate buffer was added to the medium for the cultivation of neutrophilic strains. For *A. acetoxydans* strain INE, biomass grown at both pH 3.9 and 5.0 was analyzed. Fatty acid analyses were carried out by acid hydrolysis of total cell material with 5% HCl in methanol by refluxing for 3 h, and analysis by gas chromatography, gas chromatography–mass spectrometry, following procedures described previously (Sinninghe Damsté et al., 2011), and were performed in duplicate. Intact polar lipids (IPLs) were extracted from freeze-dried biomass using a modified Bligh–Dyer procedure and analyzed by ultra-high-pressure liquid chromatography–high resolution mass spectrometry (UHPLC-HRMS) under conditions described previously (Bale et al., 2019).

### Phylogenetic Analysis

Cloning of the 16S rRNA gene was performed to determine the phylogenetic affiliation of strain INE. Total genomic DNA was extracted using the FastDNA® SPIN Kit for Soil and the FastPrep® Instrument (MP Biomedicals, Santa Ana, CA). The 16S rRNA genes were amplified with the primers set 27F-1492R (*T_a_* = 57°C) for *Bacteria* and cloned in *Escherichia coli* DH5α competent cells by using the pGEM-T vector (Promega, Madison, WI). Sequences were assembled with the DNABaser software 3.5.3 and prior to phylogenetic analysis, vector sequences flanking the 16S rRNA gene inserts were identified using the VecScreen tool (NCBI) and removed.^1^ Clone sequences were checked for chimeras (Wright et al., 2012), aligned with SINA (v1.2.11; Pruesse et al., 2012), and added to a database of over 230,000 homologous prokaryotic 16S rRNA gene primary structures by using the merging tool of the ARB program package (Ludwig et al., 2004). Sequences were then manually corrected with the alignment tool of the same software and added by parsimony to the tree generated in the *Living Tree Project* (LTP; Yarza et al., 2008). Phylogenetic reconstruction was performed using the three algorithms as implemented in the ARB package. The maximum-likelihood method was used for the generation of the consensus tree and bootstrap analysis performance. The bar indicates 10% estimated sequence divergence. The 16S rRNA gene sequence is available in the EMBL database under accession number LN551924.

### Genomic Analysis and Annotation Workflow

Genomic DNA was extracted with the MasterPure™ Gram Positive DNA Purification Kit (Epicentre, Madison, Wisconsin). Pacific Biosciences sequencing was performed on a PacBio RSII at GATC Biotech (Konstanz, Germany), and Illumina sequencing on a MiSeq sequencer (250 bp paired-end with 500 bp insert). The genome was assembled with the smrt analysis pipeline version 2.3.0 and the protocol HGAP version 3. After assembly, the taxonomy of all contigs was determined with megablast (Altschul et al., 1990) and a custom version of the LCA algorithm (Huson et al., 2007). In detail, a blast search was performed against various database of the NCBI (NCBI NT database, the draft bacteria genome database, the human genome, the protozoa database; NCBI Resource Coordinators, 2013), the Hungate 1,000 database (Seshadri et al., 2018) and the human microbiome database (Human Microbiome Project, 2012), using an e value of 0.0001. A custom script with the implementation of the LCA algorithm was used (Huson et al., 2007), with the exception that only the hits exceeding a bitscore of 50 and not deviating >10% in length from the longest hit were used to determine the taxonomy. After the taxonomy was obtained, all contigs not belonging to the family *Peptococcaceae* were discarded as contamination. A correction of the PacBio assembly was performed with Pilon 1.19 (Walker et al., 2014), and the Illumina MiSeq data, which was mapped to the assembly with bowtie2 (Langmead and Salzberg, 2012). The Illumina MiSeq data were additionally used as verification that the discarded contigs of the PacBio assembly were contaminating contigs. Average amino acid identity (AAI) was calculated with compareM v0.1.1.^2^

The annotation of the genome was performed with an early version of SAPP (Koehorst et al., 2018). Gene calling was performed with prodigal 2.6.3 (Hyatt et al., 2010). RNA genes were predicted with RNAmmer 1.2 (Lagesen et al., 2007), Aragorn 1.2.36 (Laslett and Canback, 2004), and the CRISPR Recognition tool version 1.2 (Bland et al., 2007). Protein coding genes were further annotated with InterproScan 5.19–58.0, PRIAM version March 2015 (Claudel-Renard et al., 2003), EnzDP version 1.0 (Nguyen et al., 2015), tmHMM version 2.0c (Krogh et al., 2001), and SignalP 4.1 (Petersen et al., 2011). Additional EC numbers were derived *via* Gene Ontology terms (Ashburner et al., 2000) predicted by InterproScan. Furthermore, blastp searches against the COG database (Galperin et al., 2015) and the Swissprot database (Release 06 Jul 2016; UniProt, 2014) were performed. The annotations were partially manually curated. To avoid repetition, locus ID is displayed without the locus tag “DEACI_.”

### Proteomics

Duplicate samples at each pH value of the biomass grown in reactors (pH 3.5 A + B and pH 5.0 A + B) were taken at 80% of maximum OD_600_, centrifuged, washed, and transferred to 2-ml low-binding microcentrifuge tubes (Eppendorf, Netherlands) prior to protein extraction. Around 0.1 g of wet weight pellet was resuspended in 500 ml of 4% sodium dodecyl sulfate in 100 mM Tris-HCl pH 7.6. Cells were lysed by sonication with a 3-mm tip using a Branson sonifier in six pulses of 30 s. Around 40 μg of protein was separated by gel electrophoresis (SDS-PAGE) and cut into five slices per sample to fractionate proteins. The gel slices were subjected to in-gel tryptic digestion and peptides were loaded on STAGE-tips (STop And Go Elution, C18-reversed phase SPE) for desalting and concentration.

The resulting purified and concentrated peptide mixtures were analyzed by nanoflow C18 reversed phase HPLC coupled with a maXis 4G ETD ultra-high resolution Qq-TOF mass spectrometer (Bruker Daltonics) with collision-induced dissociation (CID) as fragmentation technique. Chromatographic separation was achieved *via* a linear gradient of 7–32% acetonitrile in 90 min using 0.1% formic acid as ion pair reagent. For data analysis, a custom *Acididesulfobacillus* database with added sequences for typical contaminant proteins (e.g., skin and hair proteins) was used. For database searches, validation, and relative quantification the MaxQuant software package was used (version 1.5.0.0).^3^ A false discovery rate of 1% was tolerated at both peptide and protein level.

In total, 1,258 *Acididesulfobacillus* proteins were identified. These proteins were quantified in both replicate samples of at least one of the conditions (pH 3.9 or 5.0). Known contaminants like human keratins were excluded from these lists. Please note that no correction for multiple testing has been applied due to the number of replicates (*n* = 2) for each condition.

### Data Accessibility

All data has been uploaded to the European Nucleotide Archive under bioproject number PRJEB7665 composed by PacBio raw data (ERR3835955), Illumina MiSeq raw data (ERR665276), and genome assembly (ERZ1286184). The mass spectrometry proteomics data have been deposited to the ProteomeXchange Consortium (Deutsch et al., 2020) *via* the PRIDE (Perez-Riverol et al., 2019) partner repository with the dataset identifier PXD022508.

## Results and Discussion

### Characterization of *Acididesulfobacillus acetoxydans* gen. nov. sp. nov. strain INE

#### Morphology and Physiology

**Figure 1:**
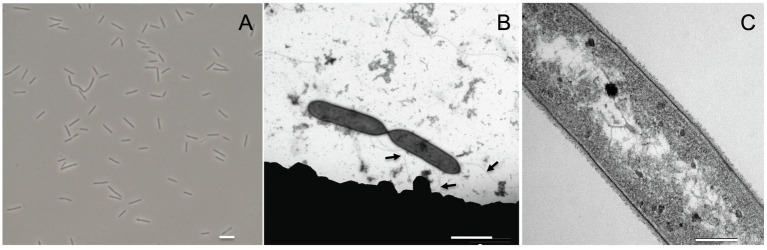
*Acididesulfobacillus acetoxydans* gen. nov. sp. nov. strain INE microscopy images: **(A)** phase contrast microscopy and **(B,C)** transmission electron microscopy. Scale bars represent 5 mm, 2 mm, and 200 nm, respectively. Arrows in **(B)** indicate flagella.

*Acididesulfobacillus acetoxydans* gen. nov. sp. nov. strain INE was isolated previously from enrichment cultures inoculated with Tinto River sediments, supplemented with lactate (5 mM) and sulfate (10 mM) at pH 4.5 (Sánchez-Andrea et al., 2013). Strain INE grew between pH 3.8 and 6.5, with a maximum specific growth rate at pH 5.0 (0.051 h^−1^). The temperature range of growth was between 25°C and 42°C, with an optimum at 30°C. Growth occurred in the presence of up to 15.3 g L^−1^ of NaCl. Cells of strain INE were single, straight rods, 4–7 μm long and *ca*. 0.6 μm wide (Figure 1A), with motility associated with observed lateral flagella (Figure 1B). Although strain INE stains Gram-negative, the double membrane characteristic of Gram-negative bacteria is absent (Figure 1C). Its Gram-positive cell wall structure was further supported by the negative KOH test and its susceptibility to vancomycin, as well as the membrane structure observed by TEM (Figure 1C).

Strain INE is a strict anaerobe as it grew only in the presence of L-cysteine, ferrous iron, titanium citrate, or sulfide as reducing agents. With sulfate as electron acceptor, strain INE utilized a wide range of compounds as electron donors (Table 1): hydrogen, complex substrates such as yeast extract, carbohydrates such as xylose, glucose, fructose, maltose, sucrose, and raffinose, a variety of C1–C4 organic acids (formate, acetate, glycolate, pyruvate, lactate, malate, fumarate, butyrate, and succinate), the amino acid L-cysteine, and alcohols, such methanol, ethanol, glycerol, and 1- and 2-propanol. Strain INE was capable of the complete oxidation of substrates to CO_2_. No growth was observed with propionate, citrate, or benzoate as electron donor. With glycerol as electron donor, strain INE used a range of compounds as electron acceptor in addition to sulfate including elemental sulfur, thiosulfate, arsenate, selenate, nitrate, and DMSO. It could not reduce ferric iron as Fe(III)NTA, sulfite, dithionate, polysulfide, perchlorate, perchloroethylene, fumarate, humic acids, chromate, molybdate, manganese, or AQDS. Strain INE was also capable of disproportionation of elemental sulfur and thiosulfate, and of fermentation of cysteine, glucose, pyruvate, and yeast extract. The only detected menaquinone of strain INE was MK-7 (100%), as also detected for the closely related *Desulfosporosinus* spp., except for *Ds. lacus* which contained up to 36% MK-5 (Ramamoorthy et al., 2006).

**Table 1:** Main characteristics differentiating strain INE from its closest phylogenetic relatives.

Name	*Acididesulfo- bacillus*	*Desulfosporosinus*				*Desulfitobacterium*	
	*A. acetoxydans* DSM 29876^T^	*Ds. acididurans* DSM 27692^T^	*Ds. acidiphilus* DSM 22704^T^	*Ds. metallidurans DSM 104464 T*	*Ds. orientis* DSM 765^T^	*D. dehalogenans* DSM 9161^T^	*D. metallireducens* DSM 15228^T^
Type strain	INE	M1^T^	SJ4^T^	OL^T^	Singapore^T^	JW/IU-DC1^T^	853-15A^T^
Isolation source	ARD sediments	AMD/ARD sediments	AMD sediments	AMD microbial mat	Soil	Freshwater pond sediment	Aquifer sediment (uranium-contaminated)
Cell size (μm)	0.7 × 5.5	0.7 × 3–5	0.8–1 × 4–7	0.5 × 2–3	1.5 × 5	0.5–0.7 × 2.5–4	
DNA G + C mol %	53.7	41.8	42.3		41.7; 42.8^1^; 45.9^2^	45	42
Quinones	MK-7 (100%)	MK-7 (98%)			MK-7		
Genome size (Mb)	4.52	4.64	4.99	5.29	5.86	4.32	3.17
Endospore position	Subterminal	Subterminal	Subterminal	Subterminal	Subterminal, paracentral or central	−	−
Motility	+	Variable	−	−	+	+	−
Gram staining	−	−	−	−	−	+	+
T range (°C)	25–42	15–40	25–40	4–37	</42		20–37
T opt (°C)	25–35	30	30	28	30–37	38	30
pH range (opt.)	3.8–6.5 (5.0)	3.8–7 (5.5)	3.6–5.6 (5.2)	4.0–7.0 (5.5)	5.6–7.4 (6.4–7)	(7.5)	(7.0)
NaCl range (%; opt)	0.5–1.5 (<0.5)	0–1.5 (0.6)	0–0.6 (1.5)	0–6 (0–0.1)	<4.5		<0.5
Catalase	−	+					
Oxidase	−	−/+	−,+		−,+		
**Electron donors**							
H_2_	+ (with 0.1 YE)	+	+	+	+	+	+
Formate	+	(+)	−	+	+	+	+
Acetate	+	−	−		−		
Pyruvate	+	+		+	+	+	+
Propionate	−	−	−	+	−		
Lactate	+	+	(+)	+	+	+	+
Malate	+	+	−	+	−		
Fumarate	+	+	−		+		
Butyrate	+	+	−		-^4^,+^1^		
Succinate	+	−	−		−		
Methanol	+	(+)	−		+		
Ethanol	+	+	−	+	+		
Glycerol	+	+	+	+	−^1^, +^3^		
Xylose	+	+	−		−		
Fructose	+	(+)	+	+	−		
Glucose	+	(+)	(+)	+	−		
**Electron acceptors**							
Sulfate	+	+	+	+	+	−	−
Sulfur	+	+	−, +^3^	−	+	+	+
Sulfite	−	−	−	+	+	+	−
Thiosulfate	+	+	+	+	+^3^**, −**^4^	+	+
DMSO	+						
Arsenate	+	+	−		−^1^^,^^2^^,^^3^, +^5^	−	
Fumarate	−	−	−	+	+	+	−
Fe(III) sol./insol.	−	Variable^3^	−		+	+	+
Nitrate	+	+	(+)^1^	+	−	+	−
PCE	−						
Se(VI)	+					+	−
Mn(IV)	−					+	+

*Desulfosporosinus acididurans* (Sánchez-Andrea et al., 2015), *Desulfosporosinus acidiphilus* (Alazard et al., 2010), *Desulfosporosinus metallidurans* (Panova et al., 2021), *Desulfosporosinus orientis* (Adams and Postgate, 1959; Campbell and Postgate, 1965; Klemps et al., 1985; Robertson et al., 2001; Ramamoorthy et al., 2006), *Desulfitobacterium dehalogenans* (Utkin et al., 1994), *Desulfitobacterium metallireducens* (Finneran et al., 2002). +, supported growth; −, did not support growth; (+), weak growth.

1 *Ramamoorthy et al. (2006)*.

2 *Robertson et al. (2001)*.

3 *Sánchez-Andrea et al. (2015)*.

4 *Adams and Postgate (1959)*.

5 *Campbell and Postgate (1965)*.

#### Phylogenetic and Genomic Analysis

**Figure 2:**
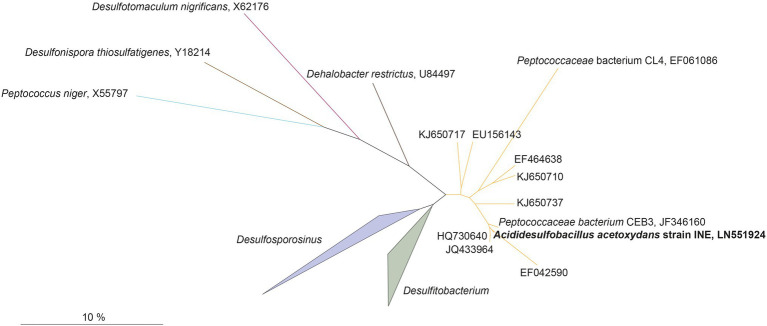
Maximum-likelihood tree showing phylogenetic affiliation of 16S rRNA gene sequences of *Acididesulfobacillus acetoxydans* (in bold) with related environmental sequences found in acidic environments, and closely related species in the *Peptococcaceae* family of the *Firmicutes* phylum. The accession numbers represent sequences retrieved from different acid drainage environments. KJ650710, KJ650737, or KJ650717: sulfidic mine tailings dumps in Botswana; EF042590: an abandoned copper mine in Odiel (Huelva, Spain); HQ730640, JQ433964: Tinto River sediments; EU156143: a thermal spring in Yellowstone National Park (WY, United States); EF464638: acidic enrichment cultures. JF346160 and EF061086: *Peptococcaceae* bacteria CEB3 and CL4. Scalebar indicates 10% sequence difference.

Genome assembly indicated the presence of one chromosome with a size of 4.52 Mbp, with a GC content of 53.65%, and 4,104 predicted protein coding sequences, of which 3,443 contained at least one protein domain, and 639 were identified as hypothetical proteins. In total 53 tRNAs and 3 full rRNA operons were identified. The phylogenetic position of the complete 16S rRNA gene sequence of strain INE (LN551924) showed that the strain is related to the *Desulfosporosinus* and *Desulfitobacterium* genera (Figure 2): the 16S rRNA gene sequence identity with its closest relatives, *Ds. meridiei* DSM 13257^T^, *Ds. auripigmenti* DSM 13351^T^ and *D. metallireducens* DSM 15288^T^ was 93.8%, 93.5%, and 93.5%, respectively. This is below the minimum similarity threshold of 94.5% for genus-delineation (Yarza et al., 2014), indicating that strain INE belongs to a new genus. This is further supported by the average amino acid identity (AAI) derived from the genome comparison of strain INE with *Desulfosporosinus* and *Desulfitobacterium* spp. (Supplementary Table S1). The highest similarity was observed with *Peptococcaceae* bacterium CEB3 (89.3%; Ňancucheo and Johnson, 2012), followed by *Ds. acidiphilus* DSM 22704 (64.4%). Based on the observed AAI values of more than 96% in most strains within one species and 62–64% within the same genus, respectively (Konstantinidis and Tiedje, 2005), AAI values also suggest that *A. acetoxydans* strain INE forms a new genus. Although the AAI with *Ds. acidiphilus* is close to the threshold of 64%, the 16S rRNA gene similarity is 92.7%, supporting the separation in a new genus. The 16S rRNA sequence identity further indicates that the novel genus *Acididesulfobacillus* encompasses the isolates CL4 and CEB3 (Jameson et al., 2010; Ňancucheo and Johnson, 2012). Although strain CL4 was formerly proposed as “*Desulfobacillus acidavidus*” (Jameson et al., 2010), it was never formally described, and current nomenclature regulations prevented us from using the proposed name. We therefore hereby propose the alternative name *Acididesulfobacillus*.

The *Desulfosporosinus* and *Desulfitobacterium* genera and the novel genus *Acididesulfobacillus* form a separate cluster from the other recognized genera and species of the family *Peptococcaceae*, order *Clostridiales* of the phylum *Firmicutes*. Members of the *Desulfosporosinus* and *Desulfitobacterium* genera are rod-shaped, Gram-positive, free-living, strictly anaerobic, and spore-forming bacteria. They display a diversity of metabolic traits relevant to bioremediation of polluted sites, e.g., reductive dehalogenation and reduction of metals such as arsenic or ferric iron, sulfur compounds, or nitrate (Spring and Rosenzweig, 2006; Alazard et al., 2010; Pester et al., 2012; Petzsch et al., 2015; Sánchez-Andrea et al., 2015; Mardanov et al., 2016). Members of *Desulfosporosinus* are known sulfate reducers in AMD environments (Nevin et al., 2003; Petrie et al., 2003; Suzuki et al., 2004; Kimura et al., 2006; Rowe et al., 2007; Cardenas et al., 2008; Lee et al., 2009; Madden et al., 2009; Senko et al., 2009; Ňancucheo and Johnson, 2014), and other low pH ecosystems such as peatlands (Hausmann et al., 2016, 2019). Three moderately acidophilic or acidotolerant *Desulfosporosinus* species were isolated so far: *Ds. acidiphilus* (pH 3.6–5.5, optimum pH 5.2; Alazard et al., 2010), *Ds. acididurans* (pH 3.8–7.0, optimum 5.5; Sánchez-Andrea et al., 2015), and *Ds. metallidurans* (pH 4.0–7.0, optimum 5.5; Panova et al., 2021).

All 16S rRNA gene amplicon sequences found in the public databases (SILVA and NCBI) that cluster together with *A. acetoxydans* strain INE were identified in AMD or ARD environments (Figure 2): in sulfidic mine tailings dumps in Botswana (pH 3.2–3.5; Korehi et al., 2014), in a stream of an abandoned copper mine in Odiel (Huelva, Spain; pH 2.5–2.75; Rowe et al., 2007), in Tinto River sediments (pH 5; Sánchez-Andrea et al., 2011) and in a thermal spring in Yellowstone National Park (pH 5.75–6.91; Hall et al., 2008). In addition, they were detected in enrichment cultures inoculated with samples from acid mine tailings (Winch et al., 2009). The two isolates included in the cluster, *Peptococcaceae* bacteria CL4 and CEB3 (Jameson et al., 2010; Ňancucheo and Johnson, 2012), were both obtained from reactors treating acid mine waters at a pH of about 2.5. The genome sequence from CEB3 is publicly available, but the two isolates were not characterized further. Interestingly, sequences of CEB3, which according to 16S rRNA gene sequence similarity corresponds to the same species as strain INE, became dominant in a reactor inoculated with an acidophilic consortium when the operating pH of the reactor was lowered, showing their predominance at more acidic conditions over other acidophilic SRB such as *Ds. acididurans* (Ňancucheo and Johnson, 2012). Its inability to grow at pH 7, combined with the lower pH optimum of strain INE (5.0), compared to *Ds. acididurans* (5.5) and *Ds. acidiphilus* (5.2; Table 1), this indicates that the SRB in this novel cluster are more acidophilic than the SRB described so far.

#### Chemotaxonomy

The lipid composition, including fatty acids (FAs), of strain INE grown at optimal pH (5.0) was compared with the type strains of the phylogenetically closest genera, *Ds. orientis* (Adams and Postgate, 1959; Stackebrandt et al., 1997) and *D. dehalogenans* (Utkin et al., 1994; Table 1). As these type strains are neutrophilic, lipid profiles were also compared with two acidophilic/acidotolerant strains from the *Desulfosporosinus* genus: *Ds. acididurans* (Sánchez-Andrea et al., 2015) and *Ds. acidiphilus* (Alazard et al., 2010).

The lipid profile of strain INE was dominated by the *iso*-C_15:0_ FA (representing almost 60%; Table 2), followed by *iso*-C_15:0_ dimethylacetal (DMA) as the second most abundant lipid. DMAs are formed upon acid hydrolysis from ether lipids containing an alk-1-enyl ether substituent at the sn-1 carbon of glycerol, so called plasmalogens (Jackson et al., 2021). Interestingly, the *iso*-C_15:0_ FA was also abundant in the acidophilic/acidotolerant strains *Ds. acididurans* (25.8%) and *Ds. acidiphilus* (16.6%), but absent in both the neutrophilic strains, *Ds. orientis* and *D. halogenans*. *Iso*-C_17:0_ and 10-methyl *n*-C_17:0_ FA and corresponding DMAs were also present in strain INE. Small amounts (i.e., 5% of total lipids) of 1-alkyl glycerol ethers with *iso*-C_15_ and 10-methyl *n*-C_17_ groups were also detected in strain INE, but saturated glycerol monoethers were absent in any of the other strains. The two most abundant FAs in *Ds. orientis* were *n*-C_16:0_ FA (35.7%) and the *n*-C_18:1_ DMA (16.4%). Both lipids are absent in strain INE and the latter is missing in all three acidophilic strains. Similarly, the abundant FAs *n*-C_16:0_ (23.6%), *n*-C_16:1_ (15.9%), and *n*-C_14:0_ (15.8%) in *D. dehalogenans*, the (neutrophilic) type strain of the second closest phylogenetic genus *Desulfitobacterium*, also showed a negligible presence in strain INE. The saturated branched-chain acid iso-C_15:0_ and the 1-alkyl glycerol ether lipids may therefore be characteristic for acidophilic SRB.

**Table 2:** Relative abundance (% of total) of fatty acids and ether lipids of *Acididesulfobacillus acetoxydans* strain INE and its phylogenetically closest relatives grown on glycerol and sulfate.

Lipid			Acidophilic/acidotolerant type strains		Neutrophilic type strains	
	*A. acetoxydans* strain INE		*Ds. acidiphilus*	*Ds. acididurans*	*Ds. orientis*	*D. dehalogenans*
	pH 3.9	pH_opt_ 5.0	pH_opt_ 5.2	pH_opt_ 5.5	pH_opt_ 6.8	pH_opt_ 7.5
**Fatty acids**						
*i*C_14_	2.5	1.1				
C_14:1_						1.4
C_14:0_	0.9		**20.4**	7.5	2.0	**15.8**
*i*C_15:1_	1.5	2.4		1.3		
*i*C_15_	**48.0**	**59.5**	**16.6**	**25.8**		
*ai*C_15_	6.5	2.4	1.4	1.5		
C_15:0_				2.4		
*i*C_16_	1.9		1.1	2.8		
C_16:1_			1.3	1.3	2.2	6.3
C_16:1_			2.6	3.9	6.3	**15.9**
C_16:0_	1.1		**32.3**	**25.9**	**35.7**	**23.6**
diMeC_15_	1.5					
10MeC_16_	2.6	1.3				
C_17:1_				3.3		
*i*C_17_	1.1	1.3	1.1	2.9		
C_17:0_				1.2		
10MeC_17_	2.1	2.5				
C_18:1_					2.4	3.8
C_18:1_					8.3	**10.3**
C_18:0_			1.4	1.5	1.1	3.8
**Sum**	**69.7**	**70.5**	**78.2**	**81.3**	**58**	**80.9**
**Sum saturated**	**68.2**	**68.1**	**74.3**	**71.5**	**38.8**	**43.2**
**Sum unsaturated**	**1.5**	**2.4**	**3.9**	**9.8**	**19.2**	**37.7**
**β-Hydroxy-fatty acids**						
C_14:0_					1.6	
C_16:1_					1.0	
C_16:0_			1.1		2.0	
C_18:1_					1.2	
C_18:1_					1.4	
**Sum**	**0**	**0**	**1.1**	**0**	**7.2**	**0**
**Dimetyl-acetals**						
*i*C_15_	4.9	**14.7**	1.4			
*i*C_16_	3.2	1.9				
C_16:1_			1.3	1.2	3.3	1.3
C_16:0_	5.4	2.1	**15.7**	7.5	9.4	6.9
C_17:1_				3.8		
*i*C_17_	4.1	5.1	1.5	4.3		
C_17:0_				1.9		
10MeC_17_		1.1				
C_18:1_					4.3	2.8
C_18:1_					**16.4**	6.7
C_18:0_			0.9		1.4	1.6
**Sum**	**17.6**	**24.9**	**20.8**	**18.7**	**34.8**	**19.3**
**1-Monoglycerol ethers**						
*i*C_15_	2.6	3.5				
*i*C_16_	1.1					
C_16:0_	2.5					
10MeC_16_	3.7					
10MeC_17_	2.3	1.5				
C_18:1_	1.0					
**Sum**	**13.2**	**5.0**	**0**	**0**	**0**	**0**

*Strains: *Acididesulfobacillus acetoxydans* strain INE at pH 3.9 and at pH 5.0; *Desulfosporosinus acidiphilus*; *Desulfosporosinus acididurans*; *Desulfosporosinus orientis*, and *Desulfitobacterium dehalogenans*. Major lipids (>10% of total) are indicated in bold*.

Analysis of the IPL of strain INE showed a high diversity of polar head groups (Supplementary Table S2), including sulfoquinovosyls (SQs), phosphoglycerols (PGs), phosphoethanolamines (PE), and diphosphatidylglycerols (DPG; commonly known as cardiolipins). Additionally, a series of glycolipids with hexosamine moieties were present. The dominant group was tentatively assigned a structure with a hexose group bound to the glycerol backbone and a hexosamine bound to this. The assignment was made based on their MS^2^ fragmentation and their accurate masses (Supplementary Figure S1; Supplementary Table S2). The second group also contained a hexosamine head group but the complete structure could not be elucidated (Supplementary Figure S1; Supplementary Table S2). Amino sugars are not routinely reported as components of polar headgroups in bacterial lipids but have been reported in a range of Archaea (Koga et al., 1993, 1998; Oger and Cario, 2013; Meador et al., 2014). Lipid-A, found in the outer-membrane of most Gram-negative bacteria, consists of two glucosamine moieties. However, the mass spectra of the hexosamine components detected in this study did not agree with published spectra of lipid A (Larrouy-Maumus et al., 2016; Crittenden et al., 2017). The IPL-bound core lipids (Supplementary Table S2) were in agreement with the hydrolysis-derived core lipid analysis (Table 2) and included diacylglycerols (DAGs) and mixed acyl/ether glycerols (AEGs; including plasmalogen lipids).

### Dominant Acid Stress Resistance Mechanisms in *Acididesulfobacillus acetoxydans*

#### Proton Consumption and Complete Organic Acid Oxidation at Optimum and Minimum pH

The pH dependence of growth of *A. acetoxydans* strain INE^T^ (pH 3.8–6.5 with pH optimum at 5.0) indicated that it is moderately acidophilic rather than acid-tolerant, since it is unable to grow at pH 7.0. As aforementioned, members of *Acididesulfobacillus* spp. were found to be more tolerant to low pH than other acidophilic/acidotolerant SRB such as *Desulfosporosinus* spp. The capacity for complete oxidation of organic acids to CO_2_ is not common in other acidophilic/acidotolerant SRB described so far (Alazard et al., 2010; Sánchez-Andrea et al., 2013, 2015) and was only recently reported to promote limited growth in *Ds. metallidurans* (Panova et al., 2021). Because of the increasing toxicity of organic acids at pH values below their pK_a_, lowering their concentration by complete oxidation likely constituted an important detoxification mechanism for *A. acetoxydans* strain INE. In order to elucidate the main strategies conferring increased robustness at low pH, we investigated this in more detail by monitoring its metabolism through quantifying proton consumption rates, and glycerol, sulfate, and acetic acid concentrations at optimum (5.0) and low (3.9) pH in pH-controlled batch bioreactors fed with glycerol.

By monitoring the acid addition required for pH control, the proton consumption rates could be calculated. A higher net consumption of protons was observed at pH 3.9 than at 5.0 (Table 3): 128 mM H^+^ g^−1^ biomass (dry weight) vs. to 58 mM H^+^ g^−1^ biomass (dry weight), respectively. Doubling times at pH 3.9 and 5.0 were 17.6 and 13.8 h, respectively. The maximum OD_600_ reached in the late exponential phase after complete oxidation of glycerol was 0.41 at pH 3.9 and 0.44 at pH 5.0, corresponding to a maximum biomass dry weight of 102 and 115 mg L^−1^, respectively (Table 3). This difference likely indicated increased energetic costs for cell maintenance at pH values below the optimum. The increased maintenance energy requirements could have been partly compensated for by the slightly larger theoretical Gibbs free energy change (Δ_r_Gʹ) for sulfate reduction with glycerol at pH 3.9 (−1186.7 kJ/mol) compared to pH 5.0 (−1142.8 kJ/mol), as calculated with Equilibrator (Flamholz et al., 2012; Table 4).

**Table 3:** Summary parameters of bioreactor cultivation of *Acididesulfobacillus acetoxydans* gen. nov. sp. nov. strain INE at pH 3.9 and pH 5.0 when grown on 5 mM glycerol and 10 mM sulfate.

	pH 3.9	pH 5.0
Doubling time *t_d_* (h)	17.6	13.8
Maximum OD_600_	0.41	0.44
C_DW,final_^*^ (mg·L^−1^)	102	115
H^+^ consumption (mM H^+^·g_DW_^−1^)	128	58

* *C_DW,final_ was calculated from OD_600_ and ratio c_DW_ (mg·L^−1^)/OD_600_ = 254.84*.

**Table 4:** Gibbs free energy of complete and incomplete oxidation of glycerol to CO_2_ and acetic acid, respectively.

Reaction equation	Δ_r_Gʹ (kJ/reaction)	
	pH 3.9	pH 5.0
**Eq. 1—Complete oxidation of glycerol to CO_2_**		
4 C_3_H_8_O_3_ + 7 SO_4_^2−^ + 14 H^+^ → 12 CO_2_ + 7 H_2_S + 16 H_2_O	−1142.8	−1099.0
**Eq. 2—Oxidation of glycerol to acetic acid**		
4 C_3_H_8_O_3_ + 3 SO_4_^2−^ + 6 H^+^ → 4 C_2_H_4_O_2_ + 3 H_2_S + 8 H_2_O + 4 CO_2_	−781.3	−776.6
**Eq. 3—Oxidation of acetic acid to CO_2_**		
C_2_H_4_O_2_ + SO_4_^2−^ + 2 H^+^ → 2 CO_2_ + H_2_S + 2 H_2_O	−90.4	−80.6

*Δ_r_G’ was calculated with Equilibrator (Flamholz et al., 2012) using concentrations of 1 mM for all reactants*.

**Figure 3:**
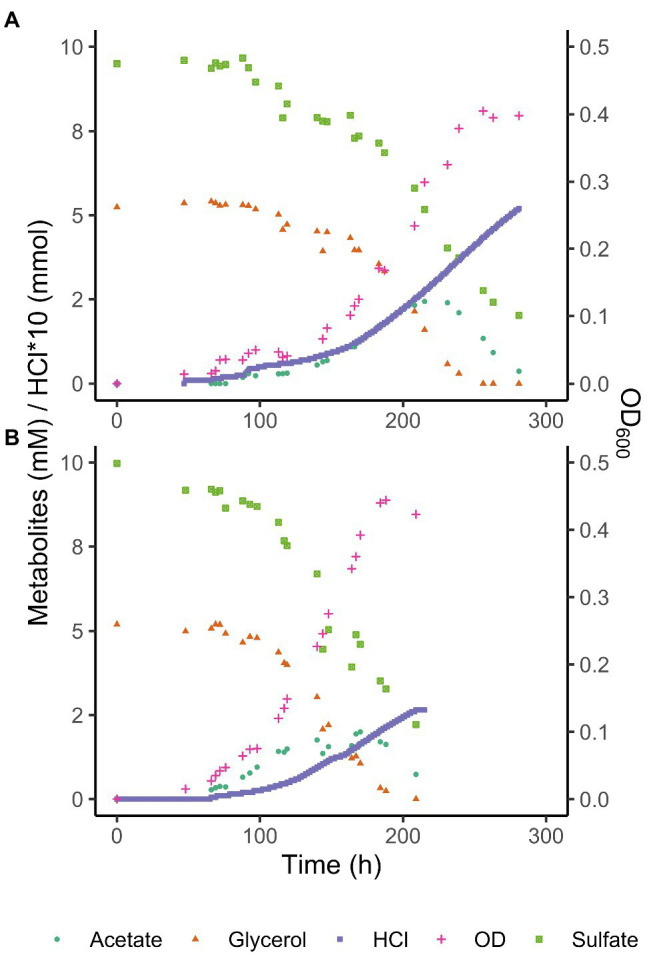
Glycerol, total acetate (undissociated + dissociated) and sulfate concentrations, and OD_600_ of *Acididesulfobacillus acetoxydans* strain INE grown in bioreactors at pH 3.9 **(A)** and pH 5 **(B)**. Concentration profiles of glycerol, sulfate, acetic acid, as well as OD_600_ and HCl addition are shown for one reactor at each pH value for clarity.

Consumption of glycerol and sulfate was accompanied by an initial increase of total acetate concentrations to 2.4 mM at pH 3.9 and 2 mM at pH 5.0, followed by its complete removal (Figure 3). Total acetic acid concentrations started to decrease before all glycerol was consumed, indicating a co-consumption phase. Biomass concentrations remained approximately constant once glycerol was completely consumed, while total acetic acid concentrations kept decreasing, indicating that under these conditions, energy derived from acetate degradation was invested in cell maintenance rather than in net growth. This was also reflected in the continued addition of acid (HCl) required to maintain constant pH after the maximum optical density (OD_600_) was reached. Overall, approximately 30% of the carbon added as glycerol was not coupled to dissimilatory sulfate reduction but used for anabolism.

The ability to use acetic acid as electron donor is advantageous in extreme environments such as AMD for several reasons. Firstly, acetic acid is a typical end product of other microorganisms such as fermenters present in AMD sediments (Sánchez-Andrea et al., 2018). The ability to metabolize a variety of organic acids, but especially a metabolic end product such as acetic acid (Table 1) increases the carbon and energy available to strain INE in nutrient-limited AMD/ARD environments. Furthermore, at pH values below their pK_a_, organic acids occur in their undissociated (protonated) form and can freely diffuse across the cell membrane, resulting in the acidification of the cytoplasm. Removal of intracellular protons is energetically costly and can be detrimental for growth. Complete oxidation of organic acids to CO_2_ prevents their accumulation in the environment, thereby lowering organic acid toxicity. For example, at the pH conditions compared in this study, maximum total extracellular acetic acid (dissociated and undissociated) concentrations reached 2.4 mM at pH 3.9 and 2.0 mM at pH 5.0 removal (Figure 3). Considering the pK_a_ of acetic acid (4.8), its extracellular concentration (undissociated) at pH 3.9 is 2.1 mM, vs. 0.7 mM at pH 5.0. The higher extracellular acetic acid concentration measured at pH 3.9 could correspond to a higher consumption of acetic acid at the optimum pH 5.

#### Metabolic Pathways for Complete Acetic Acid Oxidation Coupled to Sulfate Reduction

**Figure 4:**
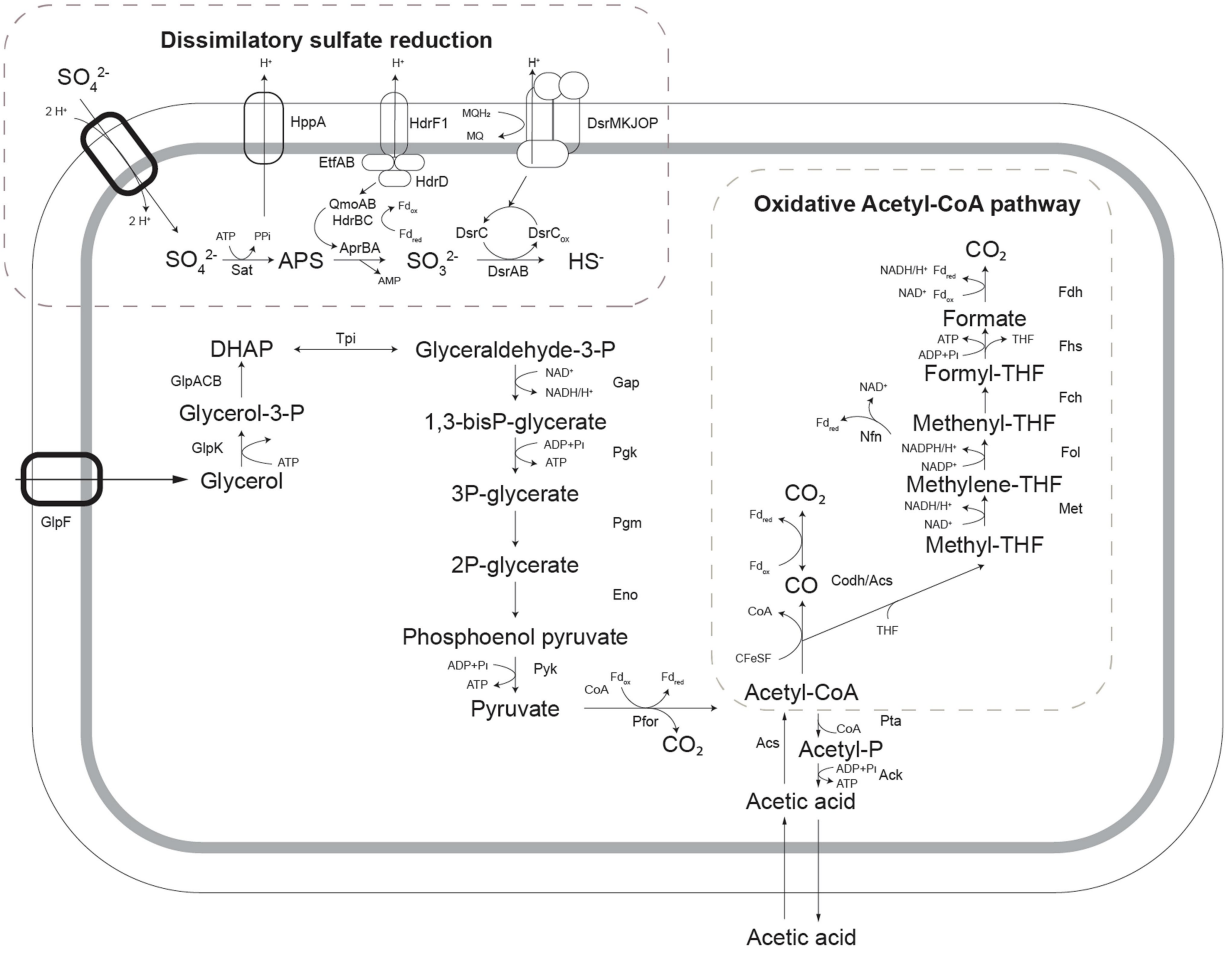
Schematic representation of pathways for glycerol oxidation and sulfate reduction as detected in the genome of *Acididesulfobacillus acetoxydans*. Sat: ATP sulfurylase, Apr: APS reductase, Dsr: dissimilatory sulfite reductase, Qmo: quinone-interacting membrane oxidoreductase complex, Hdr: heterodisulfide reductase, GlpF: glycerol transporter, GlpK: glycerol kinase, Tpi: triose phosphate isomerase, Gap: glyceraldehyde-3-phosphate dehydrogenase, Pgk: phosphoglycerate kinase, Pgm: phosphoglycerate mutase, Eno: phosphoenolpyruvate hydratase, Pyk: pyruvate kinase, Pfor: pyruvate:ferredoxin oxidoreductase, Pta: Phosphate acetyltransferase, Ack: acetate kinase, Acs: acetyl-coA synthase, Codh: carbon monoxide dehydrogenase, Met: methylene-tetrahydrofolate reductase, Fol: methylene-tetrahydrofolate dehydrogenase/methenyl-tetrahydrofolate cyclohydrolase, Fch: 5-formyltetrahydrofolate_cyclo-ligase, Fhs: Formate-tetrahydrofolate ligase/ formyl-H4F synthethase, Fdh: formate dehydrogenase, Nfn: NADH-dependent reduced ferredoxin:NADP^+^ oxidoreductase.

To enable the more informed prediction of acetotrophic potential in acidophilic SRB from (meta)genomic data, the metabolic pathway for complete oxidation of acetic acid by *A. acetoxydans* was determined through proteome analysis. The Acetyl-CoA pathway seemed to be responsible for the complete oxidation of acetic acid to CO_2_, since all of its enzymes were present in the proteome (Figure 4). Additionally, the absence of citrate synthase indicated that acetyl-CoA could not be degraded through the tricarboxylic acid (TCA) cycle, the other potential pathway for acetate oxidation. Two genes encoding bifurcating enzymes related to the reverse Acetyl-CoA pathway were detected, the NADH-dependent reduced ferredoxin:NADP^+^ oxidoreductase (*nfnAB*, 2795-96), and the cytoplasmic NADH-dependent formate dehydrogenase (*fdh*, 1374–1377). NfnAB catalyses the NAD^+^-dependent reduction of ferredoxin by NADPH, acting possibly on the NADP^+^ generated in the conversion of methylene-THF to methenyl-THF in the Acetyl-CoA pathway. The *nfnAB* genes, both encoding iron–sulfur flavoproteins, are present in a diversity of microorganisms (Wang et al., 2010), but often incorrectly annotated as sulfide dehydrogenase. The *nfnAB* genes were detected in the proteome of strain INE and were found in the same operon as two genes encoding methylene-H_4_F-DH/methenyl-H_4_F cyclohydrolase and the methenyl-H_4_F cyclohydrolase (2793-94), both part of the Acetyl-CoA pathway. The second bifurcating enzyme detected in the proteome of strain INE, the cytoplasmic NADH-dependent formate dehydrogenase (Sebban et al., 1995; Costa et al., 1997), is a selenocysteine-containing Fdh that oxidizes formate while reducing ferredoxin and NAD^+^. The Fdh detected in the genome of strain INE resembled the Fdh protein encoded in the genome of *Desulfotomaculum kuznetsovii* (Deasku_2987-2991), and consisted of a catalytic subunit (*fdhA*, 1377), two subunits for oxidoreduction of NADH/NAD^+^ (*fdhGH*, 1374), the proton-translocating quinone (*fdhE*, 1375), and the 5-formyltetrahydrofolate cyclo-ligase (*fdhH*, 1373). This Fdh is predicted to act in the Acetyl-CoA pathway (Ragsdale and Pierce, 2008), and it was found next to a gene encoding a histidine kinase (1378), suggesting that it has a regulatory function (Pereira et al., 2011). Although the complete Acetyl-CoA pathway is also present in the genome of sulfate reducers from the genus *Desulfosporosinus* (Alazard et al., 2010; Pester et al., 2012; Sánchez-Andrea et al., 2015), complete oxidation of acetic acid in moderately acidophilic SRB was so far only observed in the recently described *Ds. metallidurans*, where weak growth on acetic acid was reported. However, acetate oxidation was proposed to occur through the TCA cycle instead of the Acetyl-CoA pathway based on genome analysis (Panova et al., 2021).

Enzymes of the metabolic pathways used for oxidation of glycerol coupled to sulfate reduction were also identified in the proteome (Figure 4). Glycerol is taken up by a glycerol transporter (DEACI_1309-DEACI_1310, hereafter “DEACI_” will be omitted for simplicity) and oxidized to dihydroxyacetone-P (DHAP) by glycerol kinase (0310) and glycerol-3-phosphate dehydrogenase (1301-1303). DHAP is converted to pyruvate involving enzymes of the Embden-Meyerhoff pathway (0305-0308, 2591-2592, 3679) and subsequently to acetyl-coA by pyruvate:ferredoxin oxidoreductase (0330, 0621, 1130, 1144, 2105, 2107, 2770). Acetyl-CoA is converted to acetate through P_i_ acetyltransferase (3443) and acetate kinase (3414), or completely oxidized to CO_2_ through the reverse Acetyl-CoA pathway, discussed above. Reducing equivalents are transferred to NAD^+^ and oxidized ferredoxin. Electrons flow to sulfate through the respiratory chain, for which the key enzymes were identified. Sulfate is activated with ATP to adenosine-phosphosulfate (APS) and pyrophosphate by ATP sulfurylase (Sat, 0463). Heterodisulfide reductase (HdrF1, 0461) is predicted to receive electrons from the quinone pool and direct them to the cytoplasmic QmoAB/HdrBC complex (0456, 0457, 0470, 0471; Rabus et al., 2015), reducing APS to sulfite through the AprAB (0464, 0465) complex, as described for other SRB (Ramos et al., 2012). The sulfur in sulfite is then reduced and transferred to DsrC (0677) forming DsrC trisulfide by DsrAB (0668, 0669; Santos et al., 2015), which is finally reduced to sulfide by electrons received from the DsrMKJOP complex (0672-0676; Pires et al., 2006).

The increased specific proton consumption rate at pH 3.9 compared to pH 5.0, and the ability to oxidize organic acids to CO_2_ has important effects on the biogeochemistry of ARD/AMD environments, as it contributes to the natural attenuation of the extreme conditions. In the Tinto River, for example, the pH values can be as low as 2 in the water column (Sánchez-Andrea et al., 2012; Amils et al., 2014). Consequently, the microbial diversity in the water column of the Tinto River is generally low, and dominated by only a few species belonging to the *Acidithiobacillus*, *Leptospirillum*, and *Acidiphilium* genera (López-Archilla et al., 2001). Microbial diversity is higher in the sediments (Sánchez-Andrea et al., 2011), partly due to the wider range of physicochemical characteristics found in the different layers. As strain INE was isolated from Tinto River sediments, its detoxifying characteristics indicate that it could assist in the creation of milder conditions, where less acidophilic microorganisms can thrive. This could facilitate the higher microbial diversity found in the sediments compared to the water column, where the environment is more dynamic due to the water flow.

### Additional Stress Resistance Mechanisms at Low pH

Other possible pH homeostasis mechanisms enabling *A. acetoxydans* to grow at low pH besides acetate oxidation were also investigated. Protein abundance at optimum and minimum pH were compared to detect increased translation of proteins involved in acid stress response mechanisms at minimum pH. Protein abundances in cultures grown at optimum (5.0) and low (3.9) pH showed 1258 proteins present in at least one of the growth conditions, of which 949 were present in both. Using a value of *p* cut-off of 0.05 (student *t*-test), 30 proteins were present in at least two-fold higher abundance at pH 3.9 compared to pH 5.0, with 15 proteins unique for pH 3.9. At pH 5.0, 56 proteins were present in at least 2-fold higher abundance, of which 32 were uniquely present at pH 5.0 (Supplementary Table S3).

#### Donnan Potential and Transporters

A Donnan potential serves as a chemiosmotic barrier to the influx of protons at low extracellular pH as described for extreme acidophiles (Baker-Austin and Dopson, 2007). A key element in creating a positive membrane potential is the K^+^-transporting ATPase, encoded by the *kdpABC* operon, which was indeed detected in the genome of *A. acetoxydans* (3037-39). However, this K^+^ ATPase was not detected in the proteome of cells grown at either pH 3.9 or 5.0. Therefore, despite its apparent importance in other acidophiles (Baker-Austin and Dopson, 2007), it did not appear to play an important role in acid stress resistance of *A. acetoxydans*. Although the detection of membrane proteins such as ATPases with standard proteomics methods is difficult, and their presence cannot be completely dismissed based on these results alone, comparative transcriptomics analysis also verified the absence of the K^+^-transporting ATPase from the transcriptome (Sánchez-Andrea, unpublished data).

In addition to K^+^-ATPase, genes encoding secondary cation transporters such as Na^+^/P_i_ co-transporters (3752, 0312, 1305) and solute symporters (1779, 2169, 2179, 2830, 3466, 3909) were identified in the genome, and the corresponding proteins (1305, 2179, 3466, and 2830) were present in the proteome. Interestingly, a bile acid:Na^+^ symporter (2179) and a transporter from the major facilitator superfamily (0284) were only present at pH 3.9, indicating a potentially important role in ion transport at low pH. Build-up of excess protons in the cytoplasm can be further countered by active H^+^ export by ATPase. The proton ATPase found in strain INE (0352-0361) was present in the proteome at both pH values, but not in significantly different abundance at lower pH, suggesting that this system is not upregulated in response to increased acid stress. Antiporters such as the Na^+^/H^+^ antiporter (2829) or the H^+^/Cl^−^ exchange transporter (*clcA*, 0494) also contribute to H^+^ removal. ClcA was detected in the proteome, albeit not in significantly different abundance at lower pH.

#### Cytoplasmic Buffering Capacity

The modification of the cytoplasmic buffering capacity through a change in the metabolism of certain amino acids could mitigate acid stress (Baker-Austin and Dopson, 2007; Slonczewski et al., 2009). The decarboxylation of arginine, glutamate and aspartate, for example, releases CO_2_ and amines, and consumes protons (Slonczewski et al., 2009). This would be net H^+^-consuming when the amino acids can be taken up from the environment, and it could be speculated that the yeast extract present in the medium served as an external source of amino acids. L-Aspartate decarboxylase (2148) was found in the proteome at pH 5.0, but not at pH 3.9, indicating that this reaction does not contribute to pH homeostasis at increased proton stress. Glutamate decarboxylase (2571), catalyzing the H^+^ consuming conversion of glutamate to g-aminobutyrate (GABA; Castanie-Cornet et al., 1999), and L-arginine decarboxylase (0577), catalyzing the H^+^-consuming conversion of L-arginine to agmatine, were detected in the proteome at both conditions, albeit not at a significantly different abundance. Furthermore, glutamate decarboxylase forms the GABA shunt together with GABA aminotransferase and succinate semialdehyde dehydrogenase, which is also proposed to be involved in acid stress resistance in bacteria (Feehily and Karatzas, 2013). Through the GABA shunt, glutamate is converted to GABA by glutamate decarboxylase (2571), consuming a proton, and GABA is converted to succinate semialdehyde (SSA) by GABA aminotransferase (4212, 3258), regenerating the glutamate. Succinate semialdehyde dehydrogenase then converts SSA to succinate (3872, 3892). Glutamate decarboxylase (2571) and GABA aminotransferase (3258, 4212) were detected in the proteome of strain INE, albeit not at a significantly different abundance at pH 5.0 or 3.9. Succinate semialdehyde dehydrogenase was not detected in the proteome; however, and the role of the GABA shunt in resistance to acid stress in strain INE could, therefore, not be clearly established.

Interestingly, agmatinase (0579), converting agmatine to urea and putrescine, was detected in the proteome at both pH values. Urea may play a role in pH homeostasis through its degradation into two molecules of ammonium and one CO_2_, catalyzed by urease. This requires incorporation of 2 H^+^ and is known to play a role in acid stress resistance (Young et al., 1996). However, the gene encoding urease was not detected in the genome of strain INE. An alternative route for urea degradation could be through urea amidolyase; genes (0170, 0171) were detected in the genome, but the corresponding proteins were absent in the proteome. Urea does seem to play a role in pH homeostasis of strain INE at low pH—four out of the five enzymes of the urea cycle are present in increased abundance at pH 3.9. A carbamoyl phosphate synthase (0932) was found only at pH 3.9, and ornithine carbamoyltransferase (0934), and argininosuccinate synthase (0935) were present in twofold higher abundance. Argininosuccinate lyase (0936) was present in the proteome at both conditions in similar abundance. Arginase (0853), converting arginine to urea and ornithine and completing the urea cycle, was, however, not detected in the proteome, and the role of the urea cycle in acid stress resistance of strain INE, therefore, remains enigmatic.

#### DNA, Protein, and Lipid Repair Mechanisms

The extreme acidity and high metal concentrations in acid drainage environments increase the risk of damage to DNA, RNA, and proteins. A number of DNA, protein, and lipid repair systems were present in the proteome of strain INE. Ferroxidase (0081), linked to protection of DNA upon exposure to acid stress in *Helicobacter pylori* (Huang et al., 2010), was present in the proteome at both conditions. Several chaperones involved in protein refolding (0004, 2293, 3249, 0692, 2491, 2469, 1389, 1390) were present in the proteome, and a ferredoxin signature (3253) upstream of a chaperone (3249) was *ca*. 6-fold more abundant at pH 3.9 than at pH 5.0. An upregulation of chaperones was found to accompany growth at lower pH in many acidophiles (Jerez et al., 1988). Furthermore, six peroxiredoxins, potentially involved in lipid repair (Cárdenas et al., 2012), were detected in the proteomes at both conditions (2671, 0858, 0888, 0966, 1064, 2671, 3052). One of these (2671) was *ca*. twofold more abundant at low pH.

#### Altered Membrane Permeability

Comparison of the lipid composition in *A. acetoxydans* strain INE at pH 5.0 and at pH 3.9 showed a transition, with decreasing pH, from acyl/ether glycerol (AEG) lipids with an unsaturated ether moiety (plasmalogens) to AEG lipids with a saturated ether moiety (Table 2). This was evident in the core lipids through an increase in saturated monoalkyl glycerol ether lipids from 5.0% at pH 5.0 to 13.0% at pH 3.9 and a concomitant decrease in DMAs (from 24.9% to 17.6%). The increase in saturated ether moieties may represent an adaptation to maintain membrane homeostasis at lower pH. Increasing saturation of FAs in response to lower pH has been described some in bacterial species, but the opposite effect has been observed in others (Siliakus et al., 2017; Sohlenkamp, 2017). It should be noted that there was no change in the saturation of the diacyl glycerols (DAGs), as seen from the FAs (Table 2). With decreasing growth pH there was also a clear diversification in acyl and ether moieties (Table 2). Bacterial glycerol ether lipids likely play a role in cell resistance or adaptation to adverse environmental conditions (Grossi et al., 2015) since ether linkages are less sensitive to acid hydrolysis than ester bonds. Indeed, AEG lipids are thought to improve cell resistance to extreme external conditions, relative to DAGs (Grossi et al., 2015).

At both pH values, the *A. acetoxydans* fatty acids were dominated by branched-chain FAs (97% of FAs at pH 3.9, 100% at pH 5.0), including iso, anti-iso and mid-chain methylated FAs. Similarly, the percentage of FAs that were branch-chained was moderate to high in the other acidophilic strains, *Ds. acididurans* (26%) and *Ds. acidiphilus* (42%), while they were absent in both neutrophilic strains *Ds. orientis* and *Ds. halogenans*. In particular, the branched-chain acid iso-C_15:0_ was abundant in strain INE (48.1% at pH 3.9, 60.7% at pH 5.0) and in the acidophilic strains *Ds. acididurans* (25.8%) and *Ds. acidiphilus* (16.6). An increasing proportion of branched-chain FAs is a known adaption to maintain membrane homeostasis under external stress, and a number of extreme acidophilic bacteria have been reported to have high levels of branched-chain fatty acids (Siliakus et al., 2017).

In addition to an altered membrane FAs composition, a putative poly-gamma-glutamate (PGA) bacterial capsule synthesis protein CapA (2151) was found in 2-fold higher abundance at low pH in the proteome, providing another means to create physical separation between the cell and the acidic environment as a defense mechanism. PGA is a natural polymer that may be involved in avoiding dehydration in high salt concentrations and neutralizing pH in alkaliphiles (Ashiuchi and Misono, 2002) by forming a protective PGA capsule. Furthermore, spermidine is hypothesized to play a role in the protection against acid stress through lowering the permeability of the membrane to protons (Christel et al., 2017). Spermidine synthase (0578) was detected in both proteome sets at similar abundances, indicating spermidine could play a role in acid stress resistance of *A. acetoxydans* at both pH values tested.

## Conclusion

*Acididesulfobacillus acetoxydans* strain INE, isolated from Tinto River sediments, represents a novel species of a novel genus of moderately acidophilic SRB. The increased specific proton consumption rates at low pH (3.9) compared to optimum pH (5.0) when grown with glycerol and sulfate demonstrated the alkalinizing effect of its metabolism in acidic environments. The capacity for complete oxidation of organic acids to CO_2_ can reduce organic acid toxicity and provide increased access to organic carbon compounds in nutrient-limited acid drainage environments. Interestingly, canonical acid stress resistance mechanisms such as an inverted membrane (Donnan) potential, increased abundance of charge transporters, and cytoplasmic buffering capacity were not detected in the proteome during growth at low pH. Instead, an increased fraction of ether-bound fatty acids in the overall membrane lipid composition appeared to be an important acid stress resistance mechanism for *A. acetoxydans* during growth at pH below optimum. In addition, the increased abundance of CapA could suggest that the response to proton stress in *A. acetoxydans* resembles the response to high salinity.

### Description of *Acididesulfobacillus* gen. nov.

A.ci.di.de.sul.fo.ba.cil’lus. L. masc. adj. *acidus*, sour; N.L. masc. n. *‘Desulfobacillus’*, a (not validly published) bacterial genus name; N.L. masc. n. *Acididesulfobacillus*, an acid-loving relative of the genus ‘*Desulfobacillus*’.

Cells present sub-terminal spores and are rod-shaped, strictly anaerobic, motile, and stain Gram-negative, but lack the characteristic double membrane. The genus is closely related to *Desulfosporosinus* and *Desulfitobacterium*. The type species is *A. acetoxydans*.

### Description of *Acididesulfobacillus acetoxydans* sp. nov.

*Acididesulfobacillus acetoxydans*: a.cet.o’xy.dans. L. neut. n. *acetum*, vinegar; N.L. v. *oxydo* (from Gr. masc. adj. *oxys*, acid, sour), to oxidize; N.L. part. adj. *acetoxydans*, oxidizing acetate

Straight single rods are 4–7 μm long and about 0.6 μm wide. Motile in the exponential phase by lateral flagellation. Subterminal, oval endospores are formed in the stationary growth phase. Cells are Gram-positive that stain Gram-negative. The pH range for growth is pH 3.8–6.5, with an optimum at pH 5.0. The temperature range for growth is 25–42°C, with an optimum at 30°C. The upper limit for salt tolerance is 15.3 g L^−1^ NaCl. Strain INE uses a wide range of electron donors in the presence of sulfate such as H_2_; organic acids (formate, acetate, pyruvate, lactate, malate, fumarate, butyrate, and succinate); alcohols (methanol, ethanol, propanol, and glycerol); the amino acid L-cysteine; sugars (xylose, fructose, glucose, maltose, sucrose, and raffinose) and yeast extract or starch; but not propionate, citrate, or benzoate. Organic substrates are oxidized completely to CO_2_. Strain INE uses selenate, arsenate, nitrate, sulfate, elemental sulfur, thiosulfate, and DMSO as electron acceptors; but not iron, fumarate, PCE, perchlorate, sulfite, or dithionate. It also ferments some substrates such as cysteine, pyruvate, glucose, and yeast extract. It couples the disproportionation of sulfur and thiosulfate to growth, but not sulfite. Strain INE is susceptible to vancomycin. The predominant whole cell membrane lipids of the type strain INE at pH 5 are iso-C15:0 fatty acid (60.7%), and iso-C15:0 DMA (14.3%). Its genomic G + C content is 53.65 mol %. Phylogenetically, it is a member of the family *Peptococcaceae*, order *Clostridiales* within the Firmicutes phylum. The type strain *A. acetoxydans*, strain INE (=DSM 29876^T^ = JCM 30553^T^), was isolated from the Tinto River, Spain.

## Data Availability Statement

The datasets presented in this study can be found in online repositories. The names of the repository/repositories and accession number(s) can be found at: https://www.ebi.ac.uk/ena, PRJEB7665; https://www.ebi.ac.uk/pride/archive/, PXD022508.

## Author Contributions

IS-A and AS initiated the study and designed the experiments. IS-A and MJ performed experiments. IS-A and CG processed and analyzed the reactor, genome, and proteome data and wrote the draft manuscript. BH assembled and compared genome data. NB, JS, and WR performed membrane lipid analysis. All authors contributed to the article and approved the submitted version.

## Funding

This work was financed by ERC grants to AS (project 323009) and JS (project 694569), the research program TTW under project number 14797, which is financed by the Dutch Research Council (NWO) to IS-A, and a Gravitation grant (SIAM 024.002.002) of the Netherlands Ministry of Education, Culture and Science to AS and JS.

## Conflict of Interest

The authors declare that the research was conducted in the absence of any commercial or financial relationships that could be construed as a potential conflict of interest.

## Publisher’s Note

All claims expressed in this article are solely those of the authors and do not necessarily represent those of their affiliated organizations, or those of the publisher, the editors and the reviewers. Any product that may be evaluated in this article, or claim that may be made by its manufacturer, is not guaranteed or endorsed by the publisher.

## Footnotes

^1^ http://www.ncbi.nlm.nih.gov/VecScreen/VecScreen.html

^2^ https://github.com/dparks1134/CompareM

^3^ http://www.maxquant.org/

## Supplementary Material

The Supplementary Material for this article can be found online at: https://www.frontiersin.org/articles/10.3389/fmicb. 2022.816605/full#supplementary-material

Supplementary Table S1 AAI matrix.

Supplementary Table S2 The intact polar lipid (IPL) composition of *Acididesulfobacillus acetoxydans* gen. nov. sp. nov. grown at pH 5.0 (growth optimum) as determined by UHPLC-HRMS analysis.

Supplementary Table S3 Proteins detected at 2-fold or higher increased abundance at pH 3.9 or pH 5.0 (p-value < 0.05, student t-test). Locus tag DEACI_**** is followed by the annotation. U: unique at this condition.

Supplementary Table S4 **(A)** Example MS2 spectra of a hexosamine-hexose lipid. [M+H]+ of parent ion in MS1 = 864.604. Placement of amine group in structure is tentative. Table insert of ion assignments, mmu = milli mass unit, Δ mmu = (measured mass – calculated mass) x 1000. **(B)** Example MS2 spectra of one of the unknown hexosamine-containing lipids. [M+H]+ of parent ion in MS1 = 880.599.
