# Supplementary material for: Acetate Degradation at Low pH by the Moderately Acidophilic Sulfate Reducer Acididesulfobacillus acetoxydans gen. nov. sp. nov
Source: Front Microbiol. 2022 Mar 4;13:816605. doi: 10.3389/fmicb.2022.816605 (PMC8982180; doi:10.3389/fmicb.2022.816605)
Supplement: Supplementary Table S2 — The intact polar lipid (IPL) composition of Acididesulfobacillus acetoxydans gen. nov. sp. nov. grown at pH 5.0 (growth optimum) as determined by UHPLC-HRMS analysis. [file Table_2.docx]

Supplementary TABLE S2: The intact polar lipid (IPL) composition of *Acididesulfobacillus acetoxydans* gen. nov. sp. nov. grown at pH 5.0 (growth optimum) as determined by UHPLC-HRMS analysis.

| **Polar lipid type** | **Core lipid** | **Mass (*m/z*)** | **AEC** | **Δ mmu** | **Relative abundance** |
| --- | --- | --- | --- | --- | --- |
| SQ | AEG 15:0, O-15:0 | 770.5437 ^a^ | C_39_H_80_NO_11_S^+^ | -1.0 | 3.1 ± 0.2 |
| SQ | AEG 15:0, O-16:0 | 784.5598 ^a^ | C_40_H_82_NO_11_S^+^ | -0.5 | 1.5 ± 0.1 |
| SQ | AEG 15:0, O-17:0 | 798.5750 ^a^ | C_41_H_84_NO_11_S^+^ | -0.9 | 2.6 ± 0.2 |
| SQ | AEG 15:0, O-18:0 | 812.5908 ^a^ | C_42_H_86_NO_11_S^+^ | -0.8 | 0.8 ± 0.1 |
| SQ | DAG 15:0, 15:0 | 784.5234 ^a^ | C_39_H_78_NO_12_S^+^ | -0.5 | 0.9 ± 0.2 |
| SQ | DAG 15:0, 16:0 | 798.5396 ^a^ | C_40_H_80_NO_12_S^+^ | -0.1 | 0.4 ± 0.1 |
| SQ | DAG 15:0, 17:0 | 812.5545 ^a^ | C_41_H_82_NO_12_S^+^ | -0.7 | 0.6 ± 0.1 |
|  |  |  |  |  | **9.9 ± 0.9** |
| PG | AEG plasmalogen 15:0, O-15:1 | 696.5164 ^a^ | C_36_H_75_NO_9_P^+^ | -1.0 | 4.4 ± 0.8 |
| PG | AEG 15:0, O-15:0 | 698.5320 ^a^ | C_36_H_77_NO_9_P^+^ | -1.1 | 1.6 ± 0.1 |
| PG | AEG plasmalogen 15:0, O-16:1 | 710.5324 ^a^ | C_37_H_77_NO_9_P^+^ | -0.7 | 1.7 ± 0.1 |
| PG | AEG 15:0, O-16:0 | 712.5478 ^a^ | C_37_H_79_NO_9_P^+^ | -0.9 | 0.8 ± 0.1 |
| PG | AEG plasmalogen 15:0, O-17:1 | 724.5473 ^a^ | C_38_H_79_NO_9_P^+^ | -1.4 | 4.2 ± 0.8 |
| PG | AEG 15:0, O-17:0 | 726.5632 ^a^ | C_38_H_81_NO_9_P^+^ | -1.2 | 1.7 ± 0.1 |
| PG | AEG plasmalogen 15:0, O-18:1 | 738.5631 ^a^ | C_39_H_81_NO_9_P^+^ | -1.3 | 1.5 ± 0.1 |
| PG | AEG 15:0, O-18:0 | 740.5787 ^a^ | C_39_H_83_NO_9_P^+^ | -1.3 | 1.6 ± 0.0 |
| PG | DAG 15:0, 15:0 | 712.5111 ^a^ | C_36_H_75_NO_10_P^+^ | -1.2 | 0.3 ± 0.0 |
| PG | DAG 15:0, 16:0 | 726.5270 ^a^ | C_37_H_77_NO_10_P^+^ | -0.9 | 0.3 ± 0.0 |
| PG | DAG 15:0, 17:0 | 740.5424 ^a^ | C_38_H_79_NO_10_P^+^ | -1.2 | 1.3 ± 0.0 |
|  |  |  |  |  | **19.3 ± 2.2** |
| PE | AEG plasmalogen 15:0, O-14:1 | 634.4801 ^b^ | C_34_H_69_NO_7_P^+^ | -0.5 | 0.5 ± 0.0 |
| PE | AEG plasmalogen 15:0, O-15:1 | 648.4956 ^b^ | C_35_H_71_NO_7_P^+^ | -0.7 | 6.2 ± 0.2 |
| PE | AEG plasmalogen 15:0, O-16:1 | 662.5110 ^b^ | C_36_H_73_NO_7_P^+^ | -0.9 | 1.5 ± 0.0 |
| PE | AEG plasmalogen 15:0, O-17:1 | 676.5266 ^b^ | C_37_H_75_NO_7_P^+^ | -1.0 | 1.8 ± 0.1 |
| PE | AEG plasmalogen 15:0, O-18:1 | 690.5424 ^b^ | C_38_H_77_NO_7_P^+^ | -0.8 | 1.3 ± 0.2 |
| PE | DAG 15:0, 15:0 | 664.4904 ^b^ | C_35_H_71_NO_8_P^+^ | -0.8 | 3.0 ± 0.1 |
| PE | DAG 15:0, 16:0 | 678.5062 ^b^ | C_36_H_73_NO_8_P^+^ | -0.7 | 1.2 ± 0.0 |
| PE | DAG 15:0, 17:0 | 692.5218 ^b^ | C_37_H_75_NO_8_P^+^ | -0.7 | 3.2 ± 0.1 |
|  |  |  |  |  | **18.6 ± 0.1** |
| Hexosamine-hexose | AEG plasmalogen 15:0, O-15:1 | 848.6082 ^b^ | C_45_H_86_NO_13_^+^ | -1.2 | 5.4 ± 0.0 |
| Hexosamine-hexose | AEG 15:0, O-15:0 | 850.6241 ^b^ | C_45_H_88_NO_13_^+^ | -0.9 | 4.1 ± 1.2 |
| Hexosamine-hexose | AEG plasmalogen 15:0, O-16:1 | 862.6239 ^b^ | C_46_H_88_NO_13_^+^ | -1.1 | 0.9 ± 0.2 |
| Hexosamine-hexose | AEG 15:0, O-16:0 | 864.6395 ^b^ | C_46_H_90_NO_13_^+^ | -1.1 | 0.7 ± 0.2 |
| Hexosamine-hexose | AEG plasmalogen 15:0, O-17:1 | 876.6392 ^b^ | C_47_H_90_NO_13_^+^ | -1.5 | 1.6 ± 0.4 |
| Hexosamine-hexose | AEG 15:0, O-17:0 | 878.6552 ^b^ | C_47_H_92_NO_13_^+^ | -1.1 | 1.2 ± 0.3 |
| Hexosamine-hexose | AEG plasmalogen 15:0, O-18:1 | 890.6542 ^b^ | C_48_H_92_NO_13_^+^ | -2.1 | 2.1 ± 0.5 |
| Hexosamine-hexose | AEG 15:0, O-18:0 | 892.3670 ^b^ | C_48_H_4_NO_13_^+^ | -1.7 | 2.0 ± 0.3 |
| Hexosamine-hexose | DAG 15:0, 15:1 | 862.5886 ^b^ | C_45_H_84_NO_14_^+^ | 0.0 | 0.4 ± 0.1 |
| Hexosamine-hexose | DAG 15:0, 15:0 | 864.6039 ^b^ | C_45_H_86_NO_14_^+^ | -0.4 | 3.1 ± 0.4 |
| Hexosamine-hexose | DAG 15:0, 16:0 | 878.6194 ^b^ | C_46_H_88_NO_14_^+^ | -0.6 | 0.4 ± 0.1 |
| Hexosamine-hexose | DAG 15:0, 17:0 | 892.6346 ^b^ | C_47_H_90_NO_14_^+^ | -1.0 | 0.9 ± 0.1 |
| Hexosamine-hexose | DAG 15:0, 18:1 | 904.6346 ^b^ | C_48_H_90_NO_14_^+^ | -0.9 | 0.9 ± 0.1 |
|  |  |  |  |  | **23.7 ± 4.6** |
| Hexosamine unknown | | 864.6040 ^b^ | C_45_H_86_NO_14_^+^ | -0.3 | 1.7 ± 0.3 |
| Hexosamine unknown | | 878.6199 ^b^ | C_46_H_88_NO_14_^+^ | 0.0 | 0.2 ± 0.0 |
| Hexosamine unknown | | 890.6201 ^b^ | C_47_H_88_NO_14_^+^ | 0.1 | 0.2 ± 0.0 |
| Hexosamine unknown | | 880.5991 ^b^ | C_45_H_86_NO_15_^+^ | -0.1 | 0.9 ± 0.2 |
| Hexosamine unknown | | 906.6141 ^b^ | C_47_H_88_NO_15_^+^ | -0.7 | 0.8 ± 0.2 |
|  |  |  |  |  | **3.8 ± 0.7** |
| Lyso-DPG | AEG 15:0, plasmalogen 15:0, O-15:1 | 1074.7341 ^a^ | C_54_H_110_NO_15_P_2_^+^ | -0.4 | 2.1 ± 0.2 |
| Lyso-DPG | AEG 15:0, plasmalogen 15:0, O-16:1 | 1088.7502 ^a^ | C_55_H_112_NO_15_P_2_^+^ | 0.0 | 0.7 ± 0.0 |
| Lyso-DPG | AEG 15:0, plasmalogen 15:0, O-17:1 | 1102.7661 ^a^ | C_56_H_114_NO_15_P_2_^+^ | 0.3 | 1.0 ± 0.1 |
|  |  |  |  |  | **3.8 ± 0.3** |
| DPG | AEG 2x plasmalogens 15:0, O-15:1 | 1282.9548 ^a^ | C_69_H_138_NO_15_P_2_^+^ | 1.1 | 1.4 ± 0.1 |
| DPG | AEG 2x plasmalogens 15:0, O-15:1, 15:0, O-16:1 | 1296.9686 ^a^ | C_70_H_140_NO_15_P_2_^+^ | -0.6 | 0.9 ± 0.0 |
| DPG | AEG 2x plasmalogens 15:0, O-15:1, 15:0, O-17:1 | 1310.9838 ^a^ | C_71_H_142_NO_15_P_2_^+^ | -1.2 | 1.4 ± 0.0 |
| DPG | AEG | 1325.0011 ^a^ | C_72_H_144_NO_15_P_2_^+^ | 0.5 | 0.5 ± 0.0 |
|  |  |  |  |  | **4.6 ± 0.0** |
| Unknowns |  |  |  |  | **16.4 ± 2.4** |

*AEC = Assigned elemental composition. mmu = milli mass unit, Δ mmu = (measured mass – calculated mass) x 1000. a = [M+NH_4_]^+^ . b = [M+H]^+^. SQ = sulfoquinovosyl; PG = phosphoglycerol; PE = phosphoethanolamine; DPG diphosphatidylglycerol (commonly known as cardiolipins). DAG = diacylglycerol; AEG = mixed acyl/ether glycerol. XX:Y represent alkyl carbon number : number of double bond equivalents. An alkyl ether linkage is represented by an "O-" prefix.*
