# Supplementary material for: Acetate Degradation at Low pH by the Moderately Acidophilic Sulfate Reducer Acididesulfobacillus acetoxydans gen. nov. sp. nov
Source: Front Microbiol. 2022 Mar 4;13:816605. doi: 10.3389/fmicb.2022.816605 (PMC8982180; doi:10.3389/fmicb.2022.816605)
Supplement: Supplementary Table S3 — Proteins detected at 2-fold or higher increased abundance at pH 3.9 or pH 5.0 (p-value < 0.05, student t-test). Locus tag DEACI_**** is followed by the annotation. U: unique at this condition. [file Table_3.docx]

Supplementary TABLE S3 Proteins detected at 2-fold or higher increased abundance at pH 3.9 or pH 5.0 (p-value < 0.05, student t-test). Locus tag DEACI_**** is followed by the annotation. U: unique at this condition.

| **Locus ID \| Annotation** | **Ratio** |
| --- | --- |
| **Up at pH 3.9 (p < 0.05)** | |
| DEACI_3808\|Amidohydrolase family | U |
| DEACI_1219\|YopX-like domain, beta barrel type | U |
| DEACI_2957\|Aminotransferase class I and II | U |
| DEACI_3393\|UPF0109 protein <locus_tag> | U |
| DEACI_0929\|acetylglutamate kinase | U |
| DEACI_0150\|Hypothetical protein | U |
| DEACI_0043\|Two component system, signal transduction histidine kinase | U |
| DEACI_0431\|Putative ABC transporter | U |
| DEACI_0616\|Glutamate/leucine/phenylalanine/valine dehydrogenase signature | U |
| DEACI_1273\|Alcohol dehydrogenase, iron-type, conserved site | U |
| DEACI_2503\|diaminohydroxyphosphoribosylaminopyrimidine deaminase/glucuronate reductase | U |
| DEACI_0284\|Major facilitator superfamily transporter | U |
| DEACI_3079\|Imidazole glycerol phosphate synthase subunit HisF [hisF] | U |
| DEACI_0932\|Carbamoyl-phosphate synthase (glutamine-hydrolysing) | U |
| DEACI_2179\|arsenical-resistance protein | U |
| DEACI_1267\|Methyltransferase cognate corrinoid protein | 14.8 |
| DEACI_0220\|Putative cell wall binding repeat 2 | 8.4 |
| DEACI_3253\|3Fe-4S ferredoxin signature | 6.3 |
| DEACI_1268\|trimethylamine-corrinoid protein Co-methyltransferase | 3.4 |
| DEACI_1848\|Periplasmic binding protein-like I | 3.1 |
| DEACI_3294\|2-dehydro-3-deoxy-phosphogluconate aldolase | 3.0 |
| DEACI_3321\|Serine dehydratase-like, alpha subunit | 2.9 |
| DEACI_3250\|Domain of unknown function DUF815 | 2.4 |
| DEACI_0935\|Argininosuccinate synthase | 2.3 |
| DEACI_2359\|Bacterial translocase SecF protein signature | 2.3 |
| DEACI_1260\|Leu/Ile/Val-binding protein family signature | 2.3 |
| DEACI_1137\|Methyl-accepting chemotaxis protein (MCP) signalling domain | 2.2 |
| DEACI_0934\|ornithine carbamoyltransferase | 2.2 |
| DEACI_0227\|Putative cell wall binding repeat 2 | 2.1 |
| DEACI_0923\|Ferritin- like diiron domain protein | 2.1 |
| **Up at pH 5.0** | |
| DEACI_2761\|purine-nucleoside phosphorylase | 2.0 |
| DEACI_0532\|stage V sporulation protein T | 2.0 |
| DEACI_1328\|Uncharacterised ArCR, COG2043 | 2.0 |
| DEACI_1622\|Lipoprotein localisation LolA/LolB/LppX | 2.0 |
| DEACI_2229\|prepilin-type N-terminal cleavage/methylation domain protein | 2.1 |
| DEACI_3701\|sporulation protein, YlmC/YmxH family | 2.1 |
| DEACI_3671\|dihydroorotase | 2.2 |
| DEACI_3723\|Periplasmic binding protein domain | 2.3 |
| DEACI_2529\|Succinate dehydrogenase/Fumarate reductase transmembrane subunit | 2.3 |
| DEACI_0527\|PRC-barrel-like | 2.4 |
| DEACI_2466\|Flavin reductase like domain protein | 2.5 |
| DEACI_3992\|Putative cell wall binding repeat 2 | 2.8 |
| DEACI_4292\|UBA-like | 3.2 |
| DEACI_0833\|ferredoxin hydrogenase | 3.3 |
| DEACI_1144\|pyruvate flavodoxin/ferredoxin oxidoreductase | 3.8 |
| DEACI_4004\|Flagellar protein FliS | 4.0 |
| DEACI_4008\|Bacterial flagellin C-terminal helical region | 4.8 |
| DEACI_3249\|Hsp20/alpha crystallin family | 4.8 |
| DEACI_2496\|Hypothetical protein | 6.3 |
| DEACI_2870\|Spore coat protein CotF-like | 9.7 |
| DEACI_1833\|Hypothetical protein | 11.4 |
| DEACI_3202\|Dipicolinic acid synthetase, subunit B | 16.4 |
| DEACI_3203\|dipicolinic acid synthetase, A subunit | 17.5 |
| DEACI_0510\|Glycoside hydrolase superfamily | 34.6 |
| DEACI_2148\|aspartate 1-decarboxylase | U |
| DEACI_2885\|4Fe4S-binding SPASM domain protein | U |
| DEACI_1633\|amidophosphoribosyltransferase | U |
| DEACI_3784\|Flagellar motor switch protein FliM signature | U |
| DEACI_3666\|Orotidine-5'-phosphate decarboxylase | U |
| DEACI_0130\|Protein MutL | U |
| DEACI_2759\|serine-type D-Ala-D-Ala carboxypeptidase | U |
| DEACI_0552\|6-phosphogluconate dehydrogenase C-terminal domain-like | U |
| DEACI_4003\|Hypothetical protein | U |
| DEACI_3804\|CO dehydrogenase flavoprotein C-terminal domain | U |
| DEACI_0841\|N-acetylmuramoyl-L-alanine amidase | U |
| DEACI_4010\|NAD dependent epimerase/dehydratase, LLPSF_EDH_00030 family | U |
| DEACI_2569\|ATPase, AAA-type, core | U |
| DEACI_1838\|Gcn5-related N-acetyltransferase (GNAT) domain protein | U |
| DEACI_0871\|glutamate formimidoyltransferase | U |
| DEACI_1114\|Hypothetical protein | U |
| DEACI_0365\|uracil phosphoribosyltransferase | U |
| DEACI_1339\|Alanine racemase | U |
| DEACI_4072\|5-dehydro-4-deoxy-D-glucuronate isomerase | U |
| DEACI_2311\|SHOCT domain protein | U |
| DEACI_0982\|6-phosphogluconolactonase | U |
| DEACI_3823\|Methyl-accepting chemotaxis protein (MCP) signalling domain | U |
| DEACI_2460\|Asparagine synthase (glutamine-hydrolysing) | U |
| DEACI_1574\|acetaldehyde dehydrogenase (acetylating) | U |
| DEACI_0071\|Ferritin-related protein | U |
| DEACI_4307\|HIT-like domain protein | U |
| DEACI_0144\|Control of competence regulator ComK, YlbF/YmcA | U |
| DEACI_0416\|Ribosomal protein S18 family signature | U |
| DEACI_4066\|mannitol-1-phosphate 5-dehydrogenase | U |
| DEACI_1315\|Hypothetical protein | U |
| DEACI_0104\|RNA polymerase sigma factor 54 interaction domain protein | U |
| DEACI_2368\|Hypothetical protein | U |
