# Supplementary material for: Acetate Degradation at Low pH by the Moderately Acidophilic Sulfate Reducer Acididesulfobacillus acetoxydans gen. nov. sp. nov
Source: Front Microbiol. 2022 Mar 4;13:816605. doi: 10.3389/fmicb.2022.816605 (PMC8982180; doi:10.3389/fmicb.2022.816605)

A. Example spectra of hexosamine-hexose lipid. $[M+H]^+$ of parent ion in MS^1 = 864.60354

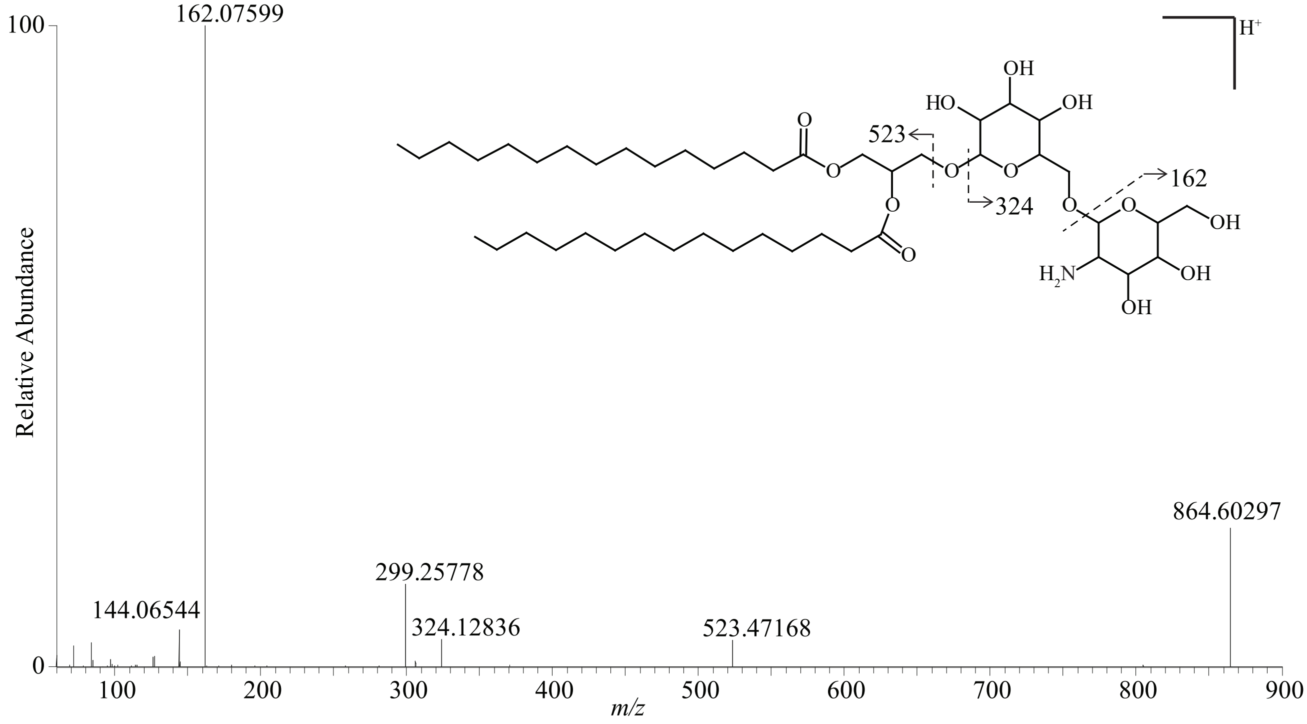

Observed mass $[M+H]^+$	Assigned elemental composition	Calculated mass	Δ mmu	Assignment
864.60354 (parent from MS^1)	$C_{45}H_{86}NO_{14}^+$	864.60428	0.7	Hexosamine-hexose-DAG
523.47168	$C_{33}H_{63}O_4^+$	523.47209	0.4	C_{30} DAG core
324.12836	$C_{12}H_{22}NO_9^+$	324.12891	0.6	Hexose-hexosamine
299.25778	$C_{18}H_{35}O_3^+$	299.25807	0.3	C_{15} fatty acid
162.07599	$C_6H_{12}NO_4^+$	162.07608	0.1	Hexosamine
144.06544	$C_6H_{10}NO_3^+$	144.06552	0.2	Hexosamine fragment (loss of H_2O)

B. Example spectra of unknown hexosamine. $[M+H]^+$ of parent ion in MS^1 = 880.5991

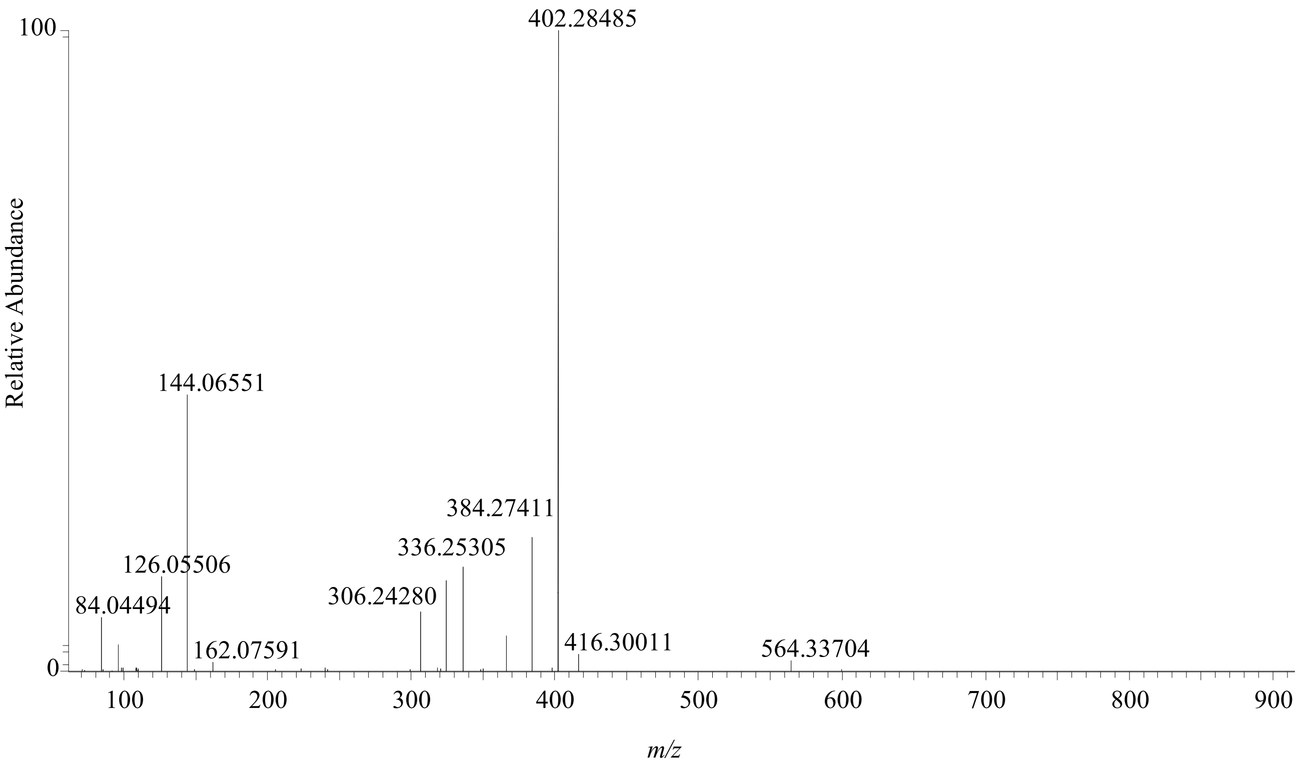

Supplement: Supplementary Table S4 — (A) Example MS2 spectra of a hexosamine-hexose lipid. [M+H]+ of parent ion in MS1 = 864.604. Placement of amine group in structure is tentative. Table insert of ion assignments, mmu = milli mass unit, Δ mmu = (measured mass – calculated mass) x 1000. (B) Example MS2 spectra of one of the unknown hexosamine-containing lipids. [M+H]+ of parent ion in MS1 = 880.599. [file Data_Sheet_1.PDF]
